# Biosynthesis of human milk oligosaccharides (HMOs) in glycoengineered human cells

**DOI:** 10.1093/glycob/cwag048

**Published:** 2026-06-22

**Authors:** Stijn Kruf, Roy J B M Delahaije, Khadra A Mohamed, Barry Schoemaker, Yoshiki Narimatsu, Henrik Clausen, Vassilis Triantis, Thomas J Boltje, Christian Büll

**Affiliations:** Department of Biomolecular Chemistry, Institute for Molecules and Materials, Radboud University, Heyendaalseweg 135, 6525 AJ, Nijmegen, The Netherlands; FrieslandCampina, Stationsplein 4, 3818 LE, Amersfoort, The Netherlands; Department of Biomolecular Chemistry, Institute for Molecules and Materials, Radboud University, Heyendaalseweg 135, 6525 AJ, Nijmegen, The Netherlands; FrieslandCampina, Stationsplein 4, 3818 LE, Amersfoort, The Netherlands; Copenhagen Center for Glycomics & Center for Glycocalyx Research, Department of Cellular and Molecular Medicine, Faculty of Health Sciences, University of Copenhagen, Blegdamsvej 3, 2200, Copenhagen, Denmark; GlycoDisplay ApS, Copenhagen, Denmark; Copenhagen Center for Glycomics & Center for Glycocalyx Research, Department of Cellular and Molecular Medicine, Faculty of Health Sciences, University of Copenhagen, Blegdamsvej 3, 2200, Copenhagen, Denmark; FrieslandCampina, Stationsplein 4, 3818 LE, Amersfoort, The Netherlands; Synthetic Organic Chemistry, Institute for Molecules and Materials, Radboud University Nijmegen, Heyendaalseweg 135, 6525 AJ Nijmegen, The Netherlands; Department of Biomolecular Chemistry, Institute for Molecules and Materials, Radboud University, Heyendaalseweg 135, 6525 AJ, Nijmegen, The Netherlands; Copenhagen Center for Glycomics & Center for Glycocalyx Research, Department of Cellular and Molecular Medicine, Faculty of Health Sciences, University of Copenhagen, Blegdamsvej 3, 2200, Copenhagen, Denmark

**Keywords:** a-lactalbumin, human milk oligosaccharides, lactose, sialyllactose, sialyltransferase

## Abstract

Human milk oligosaccharides (HMOs) are unconjugated and structurally diverse glycans synthesized in the lactating mammary gland through the stepwise action of glycosyltransferases that extend a free lactose core. Several HMOs are capped with sialic acids, including 3′-sialyllactose (3′-SL) and 6′-sialyllactose (6′-SL), that promote early-life microbiota development and contribute to immune system and neuronal functions. These health-promoting properties make sialylated HMOs attractive biomolecules for incorporation in infant nutrition and functional food products. Mammalian cell lines lack endogenous HMO production, limiting mechanistic studies of HMO biosynthesis and constraining production strategies based on human cells. Here, we developed a human cell-based strategy for the production of the two common sialyllactose isomers 3′-SL and 6′-SL in glycoengineered human embryonic kidney (HEK293) cells. We co-expressed LALBA and B4GALT1, that together form the lactose synthase complex, to introduce free lactose biosynthesis capacity into a genetically engineered human cell line without sialylation (HEK293^ΔSia^). Stable expression of either ST3GAL or ST6GAL isoenzymes in HEK293^ΔSia^ cells revealed that ST3GAL3/4/5, and especially ST3GAL5, efficiently convert lactose into 3′-SL while ST6GAL1 and ST6GAL2 produce the 6′-SL isomer. These results provide insights into the *in vivo* ability of sialyltransferase isoenzymes to use lactose as substrate. Establishing HMOs biosynthesis pathways into controllable human cell systems offers an alternative strategy for production of HMOs and provides a starting point to unlock biosynthesis of more complex HMOs in human cells.

## Highlights

- Lactose and HMO biosynthesis was installed into human cell lines.
- Genetic engineering enabled production of 3′-sialyllactose and 6′-sialyllactose.
- ST3GAL5 showed most efficient conversion of lactose into 3′-sialyllactose.
- Human cell-based production platform for complex HMOs.

## Introduction

Human milk oligosaccharides (HMOs) are complex carbohydrate oligomers composed of monosaccharides such as D-glucose, D-galactose, N-acetyl-D-glucosamine, L-fucose, and sialic acids (Bode 2012). Following lactose and lipids, HMOs are the third most abundant component in human milk with concentrations between 5–25 g/L depending on the lactation stage (Gabrielli et al. 2011; Xu et al. 2017; Mank et al. 2020). More than 200 structurally diverse HMOs have been identified and although the biological functions of many of these HMOs remain to be elucidated, several of their diverse and beneficial functions for infant development and potentially adult health are becoming increasingly clear (Bode 2012; Sprenger et al. 2022; Jin et al. 2025). The most prevalent HMOs 2′-fucosyllactose (2’-FL), 3-fucosyllactose (3-FL), 3′-sialyllactose (3’-SL), and 6′-sialyllactose (6’-SL) can be degraded by members of the gut microbiota promoting growth of beneficial *Bifidobacterium* species, cross-feeding, and production of short-chain fatty acids that support the intestinal system and systemic health (Yu et al. 2013; Lawson et al. 2020; Zhu et al. 2023; Lordan et al. 2024; Yang et al. 2025). Fucosyllactose and sialyllactose can act as decoy receptors blocking the attachment of viruses to host epithelial cells and they can exert anti-inflammatory effects and positive effects on brain function (Triantis et al. 2018; Hauser et al. 2021; Moore et al. 2021). Moreover, 3’-SL supplementation during high fat diet intake in mice was recently reported to reduce the fat mass production (Harris et al. 2020). Their prebiotic, immunomodulatory, and antimicrobial properties make HMOs important components of infant nutrition and highly attractive ingredients for functional food products (Dinleyici et al. 2023).

**Figure 1:**
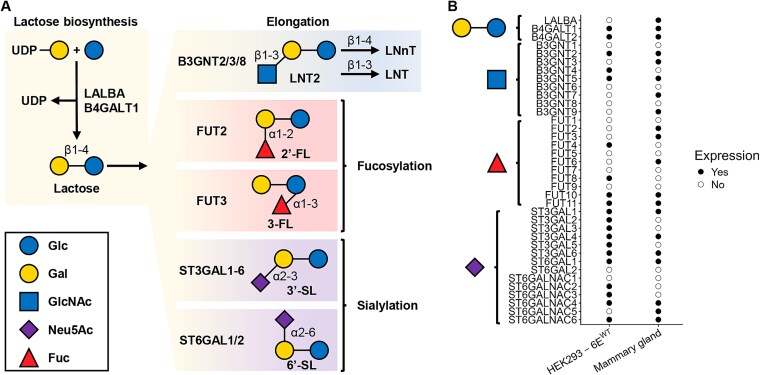
Predicted biosynthetic pathways of human milk oligosaccharides. A) Schematic drawing of the first steps of the biosynthetic pathway of HMOs and associated glycosyltransferases*.* Lactose is synthesized in mammary gland epithelial cells by the lactose synthase complex, a heterodimer composed of LALBA and B4GALT1 (Brew et al. 1968; Brodbeck U & Ebner KE, 1966; Klee WA & Klee CB, 1972). Lactose serves as the core substrate for the synthesis of diverse HMOs through sequential glycosylation reactions. Elongation, catalyzed by unspecified B3GNT family members results in the production of lacto-N-triose II (LNT2) and extended LNT2-based HMOs (Shiraishi et al. 2001; Kellman et al. 2022). Fucosylation, carried out by FUT2 and FUT3 gives rise to 2’-FL and 3-FL, respectively (Prieto 2012; Kellman et al. 2022). Sialylation, mediated by member(s) of the ST3GAL and ST6GAL sialyltransferase (ST) families, leads to the formation of 3′-sialyllactose (3’-SL) and 6′-sialyllactose (6’-SL), respectively (Kellman et al. 2022; Mohamed et al. 2024; Slater et al. 2025; Weiss GA & Hennet T 2012). B) Expression of glycosyltransferase genes in human wild type embryonic kidney (HEK293) cells and human mammary epithelial gland cells (Narimatsu et al. 2019; Twigger et al. 2022). Dot plot showing the presence or absence of transcripts encoding LALBA, B4GALTs, B3GNTs, FUTs, and STs. Filled circles denote “expressed” and empty circles denote “not expressed.”​ glycan symbols are drawn according to the symbol nomenclature for Glycans format (Varki et al. 2015).

HMOs are assembled within the Golgi apparatus of lactating mammary epithelial cells through a series of stepwise glycosylation reactions catalyzed by glycosyltransferases (GTs) (Chen 2015; Slater et al. 2025). The biosynthesis of all HMOs begins with lactose biosynthesis, a free disaccharide (Galβ1–4Glc) that is the product of lactose synthase, which links galactose to free glucose monosaccharides (Brew K & Hill RL 1975). Lactose synthase is a heterodimer consisting of a regulatory subunit LALBA (α-lactalbumin) and β1–4-galactosyltransferase 1 (B4GALT1) (Brew et al. 1968; Brodbeck U & Ebner KE, 1966; Klee WA & Klee CB, 1972). B4GALT1 is ubiquitously expressed in the different tissues and transfers galactose with a β1–4 glycosidic linkage to N-linked glycans. During lactation, B4GALT1 is upregulated in the mammary gland for lactose biosynthesis (Rajput et al. 1996; Charron et al. 1998) whereas LALBA expression is specifically induced in lactating epithelial cells upon prolactin stimulation (Turkington et al. 1968; Hannan et al. 2023). The interaction with LALBA shifts the substrate specificity of B4GALT1 from N-linked glycans to free glucose, yielding lactose (Ramakrishnan B & Qasba PK, 2001). A similar modulation of the substrate specificity by LALBA was found for the family member B4GALT2, which suggests that B4GALT2 can also play a role in lactose formation (Almeida et al. 1997). Several GTs can subsequently elongate, branch, and cap the lactose core (Fig. 1A) and despite thorough biochemical analysis over the past decades and more recent integration of omics approaches, the biosynthetic basis of many defined HMO structures is not yet fully elucidated (Kellman et al. 2022; McDonald et al. 2022; Nyquist et al. 2025; Slater et al. 2025). This is partly due to the existence of larger GT families with isoenzymes featuring redundant and competing activity as well as the lack of suitable cell systems to probe the individual contributions of specific GTs to HMO biosynthesis within the complex Golgi milieu. For example, six ST3GAL isoenzymes (ST3GAL1–6), belonging to the larger sialyltransferase (ST) family, could potentially add sialic acid to lactose to form 3’-SL (Kellman et al. 2022; Mohamed et al. 2024). It remains unclear which specific ST3GAL isoenzyme(s) utilize lactose as a substrate in the mammary gland. Unraveling the biosynthesis of HMOs and the involved GTs will unlock routes for their production in human cell-based systems mimicking the lactating mammary gland and advance studies into the structure–function relationship of complex HMOs (Bode et al. 2016).

Currently, chemical and chemoenzymatic methods are used to produce structurally defined and complex HMOs and although large-scale synthesis is technically feasible, it remains complex and labor-intensive (Agoston et al. 2019; Prudden et al. 2017; Urashima et al. 2025; Xiao et al. 2016; Zhu Y et al. 2022b). Microbial production systems have emerged for large-scale and cost-effective HMO production (Pressley et al. 2024). These systems typically employ genetically engineered microbial hosts, such as *Escherichia coli*, wherein specific GTs are introduced to enable HMO biosynthesis (Zhu Y et al. 2022a). To enhance production efficiency, competing metabolic pathways are often knocked-out, while native or heterologous pathways critical to precursor sugar and product formation are upregulated (Drouillard et al. 2006; Zhang et al. 2022). Despite the successful development of microbial HMO production systems, they are limited to simple HMOs such as 2’-FL because larger and more complex HMOs require multiple genes for human nucleotide sugar formation and GTs that are difficult to engineer into bacteria (Zeuner et al. 2019; Gan et al. 2023). Human cells endogenously express many of the HMO biosynthesis genes and mammary cells isolated from mammary tissue or differentiated from stem cells have been developed for HMO production, but they produce a heterogenous HMO mixture and it is challenging to grow them on a large scale.(Gan et al. 2023) Easy-to-grow mammalian cell lines widely used for recombinant protein production could provide an attractive platform for scalable HMO production. However, outside the lactating mammary gland, human cells do not produce significant amounts of free oligosaccharides — apart from hyaluronan or when supplemented with synthetic glycoside primers, which act as unconjugated substrate mimetics to initiate glycosylation (Dicker et al. 2014; Lugemwa FN & Esko JD 1991).

Glycoengineered human embryonic kidney cells (HEK293) have recently emerged for production of glycoproteins with defined and complex glycan structures (Narimatsu et al. 2019; Bull et al. 2020; Jaroentomeechai et al. 2024). Combinatorial gene knock-out (KO) and knock-in (KI) with precision gene editing techniques yielded libraries of genetically stable cells, with each cell expressing a specific glycan feature. These cells can be grown as suspension culture in serum-free medium and have been applied to produce recombinant glycoprotein glycoforms for functional testing (Bull et al. 2021; Nason et al. 2021; Jaroentomeechai et al. 2024). Here, we have explored the ability to introduce HMO biosynthesis pathways into these glycoengineered HEK293 cells. Overexpression of the lactose synthase complex formed by LALBA and B4GALT1 resulted in lactose production as measured by ultra-high-performance liquid chromatography with fluorescent detection (UPLC-FD). Using a panel of glycoengineered HEK293 cells, we demonstrate that expression of individual ST3GAL or ST6GAL isoenzymes can direct biosynthesis of 3′-SL and 6′-SL, and the screening of the human STs revealed the optimal isoenzymes for sialyllactose biosynthesis. Together, our study establishes glycoengineered HEK293 cells as a versatile cell-based platform for production of HMOs that may be expanded to produce complex HMOs.

## Results

### Establishing HMO biosynthesis capacity in HEK293 cells

**Figure 2:**
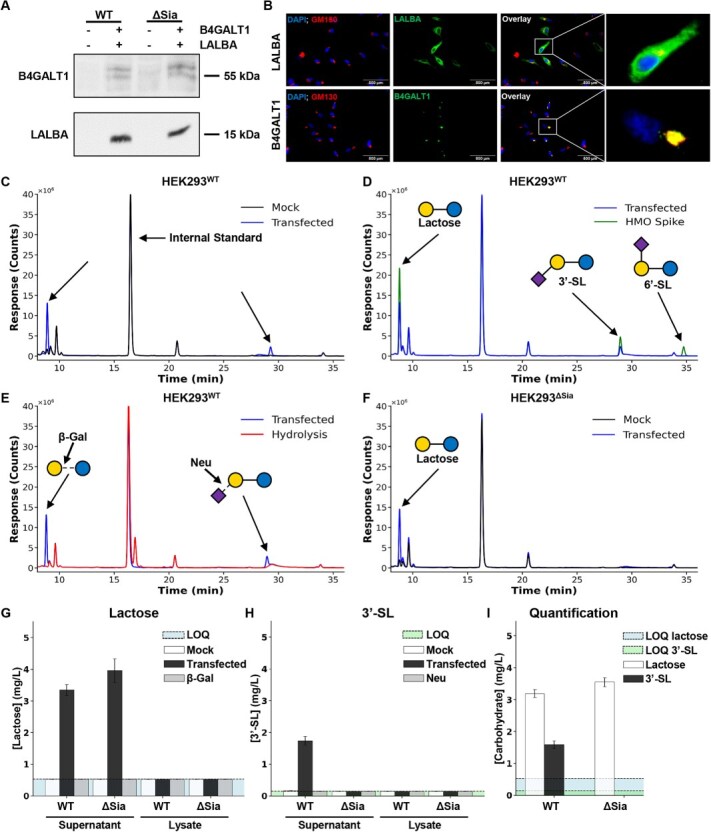
Establishing lactose and 3′-sialyllactose biosynthetic pathways in HEK293 cell lines. A) Western blot shows co-expression of 6×-his-tagged B4GALT1 (upper panel) and FLAG-tagged LALBA (lower panel) in transfected HEK293^WT^ cells and HEK293^ΔSia^ cells (knockout ST3GAL1–6, ST6GAL1/2, ST6GALNAC1–6). A representative blot of three independent experiments is shown. B) Immunocytochemistry detection of LALBA and B4GALT1 in co-transfected HeLa cells. Representative fluorescence microscopy images show nuclei (DAPI, blue), cis-Golgi (anti-GM130, red), LALBA (anti-FLAG, green, upper panel), and B4GALT1 (anti-6×his, green, lower panel). C-F) detection of HMOs in cell culture supernatant. At 72 h post-transfection with LALBA and B4GALT1, reducing carbohydrates from the culture supernatants of HEK293^WT^ cells and HEK293^ΔSia^ cells were subjected to 2-aminobenzamide labeling (2-AB) followed by ultra performance liquid chromatography analysis with fluorescent detection (UPLC-FD). Representative chromatograms of three independent experiments are shown. Arrows at peak areas indicate laminaritriose internal standard (retention time 15.9 min), lactose (retention time 8.8 min), and 3′-sialyllactose (3’-SL, retention time 29.3 min). Signals at 10, 21, and 33 min retention time are considered background signals as they are observed also in the supernatant of mock-transfected cells (C). Overlay of chromatograms from supernatants from LALBA/B4GALT1-transfected HEK293^WT^ cells spiked with or without lactose, 3’-SL, and 6′-sialyllactose (6’-SL) standards (D). Supernatants from LALBA/B4GALT1-transfected HEK293^WT^ cells were treated with β-galactosidase (β-GAL) and neuraminidase (Neu) (E). Overlay of chromatograms of supernatants derived from HEK293^ΔSia^ cells transfected with LALBA and B4GALT1 or mock-transfected control (F). G, H). Bar diagrams show lactose (G) and 3’-SL (H) concentrations (mg/L) in the supernatants and lysates of mock-transfected and LALBA/B4GALT1-transfected HEK293^WT^ and HEK293^ΔSia^ cells. Additionally, samples were treated with β-galactosidase and neuraminidase. Data are presented as mean ± standard deviation (*n* = 3) and concentrations under the limit of quantification were set to LOQ_Lactose_ (0.52 mg/L) and LOQ_3’-SL_ (0.14 mg/L). I) Bar diagram shows internal standard-based quantification with background corrected concentrations (mg/L) of lactose and 3’-SL in supernatants from LALBA/B4GALT1-transfected HEK293^WT^ and HEK293^ΔSia^ cells. Data are presented as mean ± standard deviation (*n* = 3).

To enable human cell-based production of HMOs, we explored the possibility to engineer the lactose biosynthesis pathway into HEK293 cells. LALBA expression and interaction with B4GALT1 is critical for lactose formation in the Golgi apparatus and the rate-limiting step for HMO biosynthesis (Fig. 1A) (Brodbeck U & Ebner KE, 1966; Klee WA & Klee CB 1972). Analysis of RNA-sequencing data shows that HEK293^WT^ (wild type) cells endogenously express low amounts of B4GALT1 (FPKM 6) and lack LALBA expression (Fig. 1B). Accordingly, no lactose or HMOs were detected in the culture supernatant from HEK293^WT^ cells using a detection method based on 2-aminobenzamide labeling (2-AB labelling) followed by UPLC-FD (Supplementary Fig. 1 and 2). We explored the possibility to introduce HMO biosynthesis into HEK293^WT^ cells by overexpression of LALBA and B4GALT1. Regarding its low endogenous expression in HEK293^WT^ cells and that it has been reported that B4GALT1 is upregulated for lactose biosynthesis in the mammary gland (Rajput et al. 1996; Charron et al. 1998), we co-transfected plasmids encoding for FLAG-tagged human LALBA and 6xHis-tagged B4GALT1 in a 1:1 ratio to increase the chance of efficient lactose synthase complex formation. Expression of both proteins in the cell lysates was confirmed by western blot (Fig. 2A, Supplementary Fig. 3). B4GALT1 (~54 kDa) was detected as two bands on the western blot likely representing an immature and N-glycosylated proteoform as reported by others (Khoder-Agha et al. 2019; Chen et al. 2023). To visualize their subcellular localization, we performed immunocytochemistry in HeLa cells transfected with LALBA and B4GALT1. HeLa cells were used, because of their flatter and more uniform morphology compared to HEK293 cells, allowing clearer visualization of organelles. Immunocytochemical analysis showed that LALBA was broadly distributed throughout the cell, while B4GALT1 displayed Golgi-restricted localization (Fig. 2B).

Next, we analyzed the ability of HEK293^WT^ cells co-transfected with LALBA and B4GALT1 to produce and secrete HMOs (Supplementary Fig. 4, Supplementary Table 1). At 72 h post-transfection, culture supernatants and cell lysates were collected, 2-AB labelled, and analyzed by UPLC-FD. Chromatograms of supernatant samples from LALBA/B4GALT1-transfected HEK293^WT^ cells showed an increase in peak area at retention times 8.8 and 29.3 minutes, which was absent in samples from mock-transfected HEK293^WT^ cells (Fig. 2C, Supplementary Fig. 5). To identify these reducing carbohydrates, the supernatant of LALBA/B4GALT1-transfected HEK293^WT^ was spiked with a mixture of lactose, 3’-SL, and 6’-SL. This standard-based identification revealed that lactose and 3’-SL were produced with matching retention times of 8.8 minutes for lactose and 29.3 minutes for 3’-SL (Fig. 2D, Supplementary Fig. 6). No signal was detected that matched the retention time of the 6’-SL standard (34.7 minutes) and no other HMO species were detected. The peaks corresponding to lactose and 3’-SL were sensitive to β-galactosidase and neuraminidase treatment of the supernatant, respectively, confirming their identity (Fig. 2E, Supplementary Fig. 7). Neuraminidase and galactosidase treatment resulted in the formation of an unidentified peak at 17 minutes retention time. Formation of 3′-SL was anticipated based on the expression levels of ST3GAL isoenzymes in HEK293^WT^ cells that potentially all can use lactose as substrate (Fig. 1A and B). Expression of other GTs known or predicted to form 6’-SL (ST6GAL1/2), fucosyllactose (FUT2/3), or elongated LNT and LNnT structures (B3GNT2/3/8) is low or absent in HEK293^WT^ cells and these HMOs may not be formed or their concentrations are below the detection limit.

To further corroborate the cell-based production of 3’-SL, we transfected LALBA and B4GALT1 into previously generated HEK293^ΔSia^ cells that lack endogenous sialylation capacity (Narimatsu et al. 2018; Bull et al. 2021). This stable isogenic cell line was generated by a combinatorial CRISPR-Cas9-mediated gene knock-out of human ST3GAL1–6, ST6GAL1/2, and ST6GALNAC1–6 and thus is devoid of sialic acid capping. Expression of LALBA and B4GALT1 was similar to HEK293^WT^ cells and the culture supernatant of HEK293^ΔSia^ cells contained lactose, but lacked 3’-SL (Fig. 2A and F, Supplementary Fig. 8–10). Together, these results demonstrate that reconstitution of the lactose synthase complex in HEK293 cells is sufficient to establish de novo lactose production and enables endogenous STs to generate 3′-SL, thereby functionally installing the first step of HMO biosynthesis in a human cell line.

### Quantification of lactose and 3’-SL levels

To determine the concentration of HMOs in the culture supernatants and lysates collected from HEK293^WT^ and HEK293^ΔSia^ cells transfected with LALBA/B4GALT1, standard-based quantification using laminaritriose as internal standard and calibration standards of lactose and 3’-SL was performed by UPLC-FD. With this approach, we obtained recovery rates between 85–110% for standards spiked into the culture supernatants and cell lysates (Supplementary Fig. 11 and 12, Supplementary Data Set 1). To assess intermediate precision of the analysis, supernatants from HEK293^WT^ cells transfected with LALBA/B4GALT1 were measured on four separate days and showed relative standard deviations (RSDs) of lactose and 3’-SL concentrations between 5.5% and 3.8%, respectively (Supplementary Fig. 13, Supplementary Data Set 1). This validates that lactose and 3’-SL were extracted efficiently from the samples and that our UPLC-FD method has low variability.

Lactose concentrations in the culture supernatant of mock-transfected HEK293^WT^ cells and HEK293^ΔSia^ cells were below the limit of quantification (LOQ_Lactose_ = 0.52 mg/L, Supplementary Data Set 1) and reached up to 8.2 times of the LOQ_Lactose_ after overexpression of LALBA/B4GALT1 (Fig. 2G). The addition of β-galactosidase to the culture supernatants rendered signals below the limit (LOD_Lactose_ = 0.009 mg/L, Supplementary Data Set 1). 3’-SL levels in the supernatant of LALBA/B4GALT1-expressing HEK293^WT^ cells were up to 14.3 times of the LOQ_3’-SL_ (0.14 mg/L, Supplementary Data Set 1), but below the LOD_3’-SL_ (0.002 mg/L, Supplementary Data Set 1) after treatment with neuraminidase and in HEK293^ΔSia^ cells (Fig. 2H). Interestingly, lactose and 3’-SL were below the LOD in the lysates of LALBA/B4GALT1-transfected HEK293^WT^ and HEK293^ΔSia^ cells, arguably reflecting HMO synthesis in the ER/Golgi apparatus and subsequent secretion (Fig. 2G and H, Supplementary Fig. 12 and 14). Calculations of the HMO concentrations in the culture supernatants of LALBA/B4GALT1-transfected cells corrected for the respective mock-transfected peak area revealed lactose concentrations of 3.19 mg/L in HEK293^WT^ cells and 3.55 mg/L in HEK293^ΔSia^ cells, and 3’-SL concentrations of 1.59 mg/L for HEK293^WT^ cells (Fig. 2I).

### Contribution of ST3GAL1–6 to 3’-SL biosynthesis

**Figure 3:**
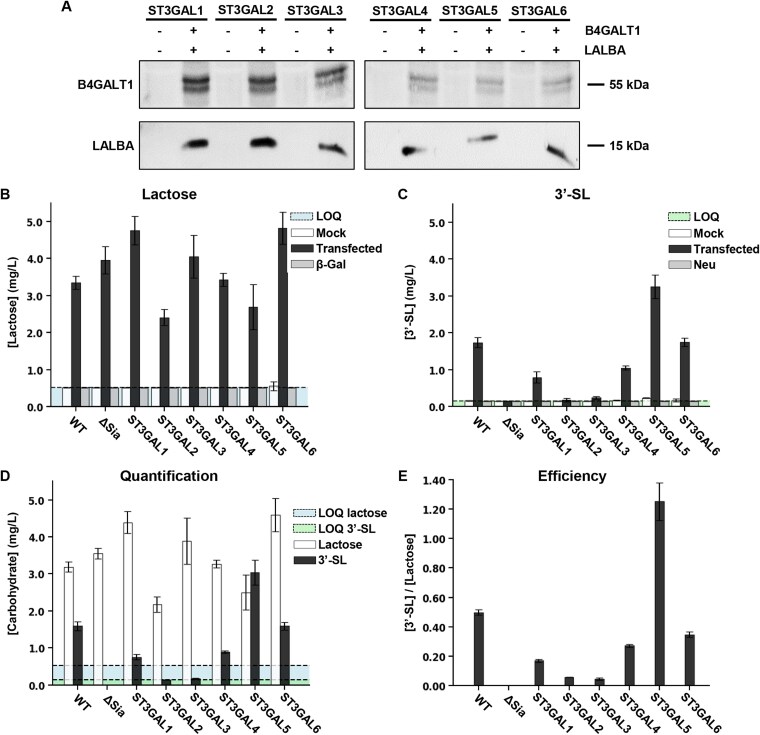
Individual contribution of ST3GAL1–6 to 3’-SL biosynthesis. A) Representative western blots show expression of FLAG-tagged LALBA and 6×-his-tagged B4GALT1 in co-transfected HEK293^KI ST3GAL1–6^ cells. B, C) bar diagrams show lactose and 3′-sialyllactose concentrations (mg/L) in supernatants of mock-transfected and LALBA and B4GALT1-transfected HEK293^WT^, HEK293^ΔSia^, and individual HEK293^KI ST3GAL1–6^ cells treated with or without β-galactosidase (β-GAL) or neuraminidase (Neu). Data are presented as mean ± standard deviation (*n* = 3) and limits of quantification for lactose (LOQ_Lactose_ = 0.52 mg/L) and 3’-SL (mg/LLOQ_3’-SL_ = 0.14 mg/L) were set. D) Bar diagram shows background corrected concentrations (mg/L) of lactose and 3’-SL in supernatants of HEK293^WT^, HEK293^ΔSia^, and HEK293^KI ST3GAL1–6^ cells transfected with LALBA/B4GALT1 calculated from internal standard-based quantification. Data are presented as mean ± standard deviation (*n* = 3). E) Bar diagram shows conversion factors indicating the efficiency for the conversion of lactose into 3’-SL for LALBA and B4GALT1-transfected HEK293^WT^, HEK293^ΔSia^, and HEK293^KI ST3GAL1–6^ cells. The conversion factor was calculated by dividing the 3’-SL concentration by the lactose concentration of each individual cell line. Data are presented as mean ± standard deviation (*n* = 3).

HEK293^WT^ cells endogenously express a mix of ST3GAL1–6 and it is not fully known which of these isoenzymes can catalyze 3’-SL biosynthesis in vivo (Kellman et al. 2022; Mohamed et al. 2024; Weiss GA & Hennet T, 2012). The HEK293^ΔSia^ cell line serves as blank background to reconstitute expression of individual STs, allowing to evaluate their activity towards lactose as substrate and efficiency of sialyllactose formation in the absence of redundant and competing STs. We have previously generated a panel of isogenic cell lines based on the HEK293^ΔSia^ cell line through targeted gene KI, with each cell expressing only a single ST3GAL isoenzyme with comparable levels (Bull et al. 2021). The six HEK293^KI ST3GAL1–6^ cell lines were co-transfected with LALBA and B4GALT1 and the protein expression at 72 h post-transfection was similar between the cell lines (Fig. 3A, Supplementary Fig. 15). UPLC-FD analysis of the culture supernatants and cell lysates showed that all HEK293^KI ST3GAL1–6^ cell lines secreted lactose and 3’-SL (Fig. 3B and C, Supplementary Fig. 16-18), with β-galactosidase and neuraminidase treatment efficiently decreasing lactose and 3’-SL levels below the LOD, confirming the formation of lactose and 3’-SL respectively. Lactose concentrations were in the range of 2.17 to 4.59 mg/L, which was comparable to 3.19 mg/L in HEK293^WT^ cells. Moreover, 3’-SL levels in the supernatants ranged from 0.17 to 3.04 mg/L (Fig. 3D). Supernatant from HEK293^KI ST3GAL5^ cells contained the highest 3’-SL concentration (3.04 mg/L), almost twice as high as the HEK293^WT^ cells (1.59 mg/L) (Fig. 3D). Cells reconstituted with ST3GAL6 yielded 3’-SL concentrations similar to HEK293^WT^ cells and ST3GAL1 and ST3GAL4 expression produced approximately half the amount. HEK293^KI ST3GAL2^ and HEK293^KI ST3GAL3^ cells transfected with LALBA and B4GALT1 showed a detectable increase in 3’-SL compared to mock-transfected cells, but the concentrations remained close to the LOQ_3’-SL_. In line with findings from HEK293^WT^ and HEK293^ΔSia^ cells, neither lactose nor 3’-SL was detectable in lysates of the HEK293^KI ST3GAL1–6^ cell lines (Supplementary Data Set 1).

To assess the efficiency of each ST3GAL isoenzyme in converting lactose to 3’-SL, we calculated a conversion factor as the ratio of the mean concentration 3’-SL to lactose (Fig. 3E). The conversion factor of HEK293^WT^ cells was 0.50 and HEK293^KI ST3GAL5^ cells exhibited the highest conversion factor (1.25), followed by KI cells with ST3GAL6 (0.35), ST3GAL4 (0.27), and ST3GAL1 (0.17). HEK293^KI ST3GAL3^ cells showed only very low conversion (0.04) and the conversion factor for HEK293^KI ST3GAL2^ cells could not be determined due to a 3’-SL concentration below the LOQ_3’-SL_. Considering potential differences in proliferation rates and transient expression levels following LALBA/B4GALT1 transfection, we cannot definitively rank the relative efficiencies of individual ST3GAL isoenzymes in 3′-SL biosynthesis. However, since cell numbers, growth conditions, and transfection efficiencies were comparable across the isogenic cell lines, our data strongly indicate that ST3GAL5 converts lactose into 3’-SL most efficiently.

### Contribution of ST6GAL1/2 to 6’-SL biosynthesis

**Figure 4:**
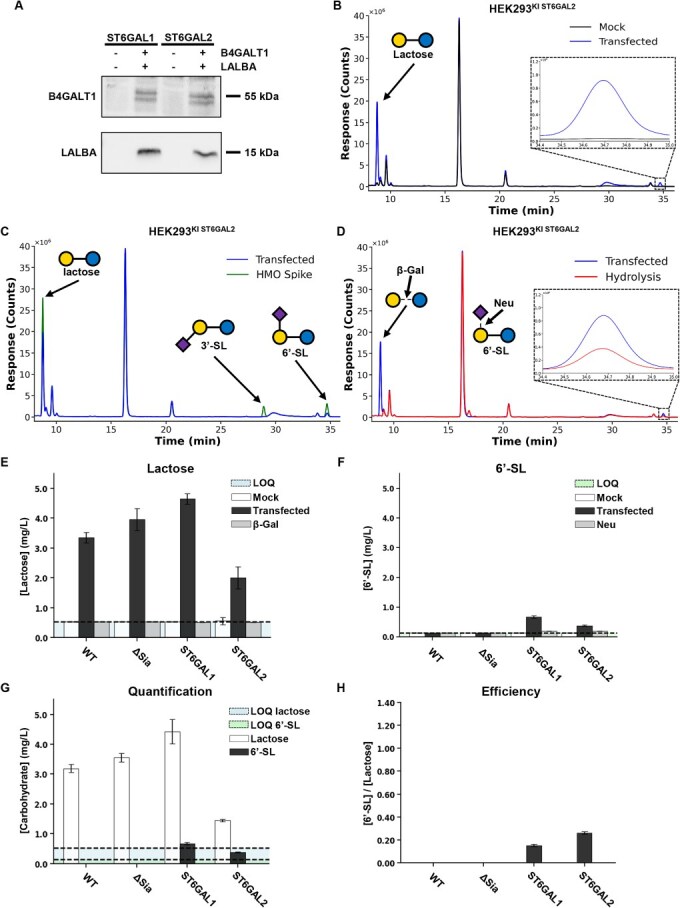
Formation of 6’-SL in HEK293^KI ST6GAL1/2^ cells. A) Western blot shows expression of FLAG-tagged LALBA and 6×-his-tagged B4GALT1 in HEK293^KI ST6GAL1/2^ cells. B-D) representative overlay chromatograms show lactose formation and 6′-sialyllactose (6’-SL) formation in LALBA and B4GALT1-transfected HEK293^KI ST6GAL2^ cells. Increased areas under the curve at retention times 8.8 min and 34.7 min in LALBA/B4GALT1-transfected HEK293^KI ST6GAL2^ cells represent the formation of lactose and 6’-SL, respectively (B). Standard-based identification using supernatant spiked with a mix of lactose, 3′-sialyllactose (3’-SL), and 6’-SL shows co-elution of the peaks at 8.8 and 34.7 minutes, verifying the identity of lactose and 6’-SL in HEK293^KI ST6GAL2^ after LALBA and B4GALT1 overexpression (C). Peaks corresponding to lactose and 6’-SL are affected by treatment of the supernatant with β-galactosidase (β-GAL) and neuraminidase (Neu) (D). E, F) bar diagrams show lactose (E) and 6’-SL (F) concentrations (mg/L) in supernatants of mock-transfected and LALBA and B4GALT1-transfected HEK293^WT^, HEK293^ΔSia^, and HEK293^KI ST6GAL1/2^ cells treated with or without β-galactosidase and neuraminidase. Data are presented as mean ± standard deviation (*n* = 3) and concentrations under the limit of quantification for lactose (LOQ_Lactose_ = 0.52 mg/Lmg/L, LOQ_6’-SL_ = 0.14 mg/L) were set. G) Bar diagram shows background corrected concentrations (mg/L) of lactose and 6’-SL in supernatants of HEK293^WT^, HEK293^ΔSia^, and HEK293^KI ST6GAL1/2^ cells transfected with LALBA/B4GALT1. Values were calculated from internal standard-based quantification and are presented as mean ± standard deviation (*n* = 3). H) Bar diagram depicts conversion factors of 6’-SL formation for HEK293^WT^, HEK293^ΔSia^, and HEK293^KI ST6GAL1/2^ cells. The conversion factor was calculated by dividing the 6’-SL concentration by the lactose concentration of each individual cell line. Data are presented as mean ± standard deviation (*n* = 3).

ST6GAL1 and ST6GAL2 can both potentially catalyze the conversion of lactose into 6′-SL. HEK293^WT^ cells only express low amounts of ST6GAL1 and we could not identify 6’-SL production after LALBA and B4GALT1 expression, suggesting that higher enzyme levels are needed (Fig. 2C and D, Supplementary Fig. 5 and 6). We investigated whether this 6’-SL is produced by ST6GAL1 or ST6GAL2 KI cell lines derived from the HEK293^ΔSia^ cell line (Bull et al. 2021). Both cell lines expressed LALBA and B4GALT1 and subsequent analysis of the supernatant showed an increase in peak area at 8.8 and 34.7 minutes compared to the mock-transfected condition (Fig. 4A and B, Supplementary Fig. 19 and 20). Spiking with lactose, 3’-SL, and 6’-SL standards revealed that lactose and 6’-SL were produced with matching retention times, 8.8 minutes for lactose and 34.7 minutes for 6’-SL (Fig. 4C, Supplementary Fig. 21). The peaks corresponding to lactose and 6’-SL were sensitive to β-galactosidase and neuraminidase treatment of the supernatant, respectively, confirming the identity of lactose and 6’-SL (Fig. 4D-F, Supplementary Fig. 22). The partial hydrolysis of 6′-SL can potentially be explained by the preference of the used neuraminidase for α2–3 linkages over α2–6 linkages (Li J & McClane BA 2014; Roggentin et al. 1995). Quantification based on the internal standards showed that the secreted 6′-SL concentrations were 0.67 mg/L for HEK293^KI ST6GAL1^ cells and 0.38 mg/L for HEK293^KI ST6GAL2^ cells (Fig. 4G). The conversion factor for HEK293^KI ST6GAL2^ cells (0.26) was slightly higher compared to HEK293^KI ST6GAL1^ cells (0.15) (Fig. 4H). This demonstrates that ST6GAL1 and ST6GAL2 both can use lactose as substrate to form 6’-SL and that there was no clear difference in efficiency between these sialyltransferases.

## Discussion

**Figure 5:**
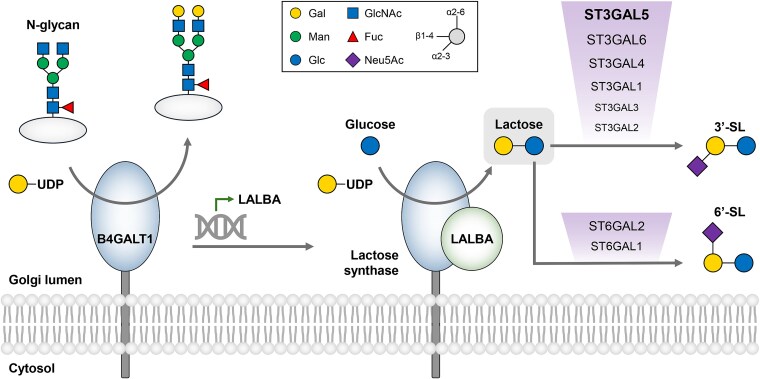
Proposed mechanism for the biosynthesis of lactose, 3′-sialyllactose and 6′-sialyllactose in glycoengineered HEK293 cells. When LALBA is absent, B4GALT1 catalyzes the transfer of galactose (GAL) from its activated sugar donor UDP-galactose (UDP-GAL) to N-acetylglucosamine (GlcNAc) residues on N-glycans forming β1–4 linkages. When LALBA is present, it forms a heterodimeric complex with B4GALT1 called lactose synthase. Lactose synthase uses free glucose as a substrate, and the transfer of galactose from UDP-GAL forms lactose through a β1–4 linkage (Ramakrishnan B & Qasba PK, 2001). Lactose can subsequently undergo α2–3 or α2–6 sialylation yielding 3′-sialyllactose (3’-SL) and 6′-sialyllactose (6’-SL), respectively. Results from our study suggest that ST3GAL5 followed by ST3GAL6 and ST3GAL4 are most efficient in 3’-SL formation *in vivo*. Both ST6GAL1 and ST6GAL2 are able to convert lactose into 6’-SL.

HEK293 cells, like most other human cell lines, lack endogenous capacity for production of HMOs due to the lack of lactose synthase expression. We were able to introduce de novo lactose biosynthesis through overexpression of LALBA and B4GALT1 (Fig. 5). Endogenous expression of GTs responsible for sialylation, fucosylation, elongation, and branching of HMOs determines which HMO structures are formed from lactose. We found that HEK293^WT^ cells secrete 3’-SL, which can be explained by the expression of ST3GAL1–6 isoenzymes. Based on the low/medium expression of ST6GAL1, FUTs, and B3GNTs, we expected to potentially find other HMOs as well. Recent work by Noda et al. (Noda et al. (2026)) confirmed that LALBA and B4GALT1 overexpressing HEK293^WT^ cells predominantly secrete 3’-SL. Using mass spectrometry analysis of concentrated supernatant samples, they detected additional HMOs with low abundance including 2’-FL, LNT/LNnT, and LSTa/c/d. This suggests that other HMOs may be produced also in our HEK293 system, but that concentrations in the culture supernatant were below the UPLC-FD detection limit. Concentration of samples prior to analysis potentially allows detection of additional HMOs and further rational genetic engineering and overexpression of GTs can increase yields and more selective production of specific HMOs.

Expressing individual ST isoenzymes in the HEK293^ΔSia^ cell line revealed their ability to convert lactose into 3’-SL and 6’-SL. It should be noted that all ST KIs are homozygous and were all made into the same *AAVS1* locus so that ST expression levels are comparable. Also, LALBA and B4GALT1 expression levels were comparable across the KI cell lines and HMO production levels were reproducible. Thus, although we cannot rule out that there is some variability between the KI cell lines, our analysis allows comparison of the lactose conversion factor between different ST isoenzymes. We found that each ST3GAL isoenzyme was able to produce 3’-SL, but clearly ST3GAL5 produced the highest levels. ST3GAL5 (GM3 synthase) is involved in the biosynthesis of sialogangliosides by adding α2–3-linked sialic acids to lactosylceramide (Galβ1–4Glcβ-ceramide) (Ishii et al. 1998; Kono et al. 1998; Kapitonov et al. 1999; Boccuto et al. 2014). Lactosylceramide consists of a lactose moiety linked to a ceramide lipid molecule and thus is highly similar to lactose, which might explain the high lactose-to-3’-SL conversion factor for ST3GAL5. It should be noted that ST3GAL5 is probably not involved in natural 3’-SL biosynthesis, because it is not/low expressed in human lactating mammary glands. Moreover, ST3GAL4 has been demonstrated to be the main ST for 3’-SL in mice and ST3GAL6 has been associated with 3’-SL production in human and bovine milk (Fuhrer et al. 2010; Liu et al. 2019; Kellman et al. 2022; Spreckels et al. 2025). Likewise, we found that both ST6GAL1 and ST6GAL2 can form 6’-SL, but ST6GAL2 is not expressed in the human mammary gland and ST6GAL1 has been shown to be responsible for 6’-SL synthesis in mice (Dalziel et al. 2001; Kellman et al. 2022).

The employed glycoengineered HEK293 cells lines recapitulate reported ST activities for 3’-SL and 6’-SL biosynthesis in the mammary gland and moreover reveal other, potentially more potent, ST isoenzymes that use lactose as substrate. The identification of ST3GAL5 and ST6GAL2 as catalysts for *in vivo* lactose conversion into 3’-SL and 6’-SL, respectively, may facilitate the development of human cell-based HMO production systems. Interestingly, in vitro studies using secreted murine ST3GAL5 and human ST6GAL2 reported no lactose sialylation for these STs (Kono et al. 1998; Takashima et al. 2002). This suggests that embedding into the Golgi environment influences the substrate scope of STs, possibly via multimerization between GTs and presence of co-factors (Stern et al. 2000; Kellokumpu et al. 2016). How biosynthetic steps for HMO biosynthesis are organized on the Golgi level should be further investigated as this is critical for understanding HMO production and regulation. Our genetic glycoengineering approach with ability to delete GTs and to express specific sets or individual GTs may be useful to dissect HMO biosynthesis in the Golgi and to probe the collaborative and competitive functions of GTs (Narimatsu et al. 2021). We have shown that this approach allows us to selectively steer HMO biosynthesis towards either 3’-SL or 6’-SL and this could be a starting point for future glycoengineering strategies to install the biosynthetic pathway of other defined, more complex HMOs. For example, expression of B3GNT family members can be explored to induce β1–3 GlcNAc elongation of the lactose core, which is the molecular basis for type 1 and type 2 HMOs.

In this study, we have used transient overexpression of LALBA and B4GALT1 and achieved lactose and sialyllactose concentrations in the mg/L range in the supernatants 72 h post-transfection. Sialyllactose concentrations in human milk are approximately 100–200 times higher (Soyyilmaz et al. 2021), but our study is an important proof-of-concept for HMO production in human cell lines. Arguably, the yields can be enhanced through stable expression of lactose synthase and optimized growth and supernatant harvest conditions. In addition, the metabolic flux through the HMO biosynthesis pathway may be enhanced via altering expression of genes involved in monosaccharide metabolism, nucleotide sugar activation, and transport into the Golgi apparatus (Moons et al. 2019; Freeze et al. 2022). However, many of the metabolic pathways and transporters in the lactating mammary gland are not fully understood. For example, mammary glands express several glucose transporters including GLUT1 that can relocate from the plasma membrane to the Golgi membrane to transport glucose into the Golgi lumen for lactose formation (Nemeth et al. 2000; Zhao 2014), GLUT8 also plays a role in Golgi glucose transport and can modulate lactose levels (Villagran et al. 2019). Further insights into the metabolic bottlenecks of HMO biosynthesis can assist engineering of the HEK293 cells to boost HMO yields. Although microbial platforms such as *E. coli* and *Saccharomyces cerevisiae* enable large-scale production of simple HMOs (e.g. 2′-FL and 3′-SL) (Yu et al. 2018; Zhang et al. 2019; Lv et al. 2024; You et al. 2024), the HEK293 cell system has two main advantages. First, these cells endogenously express many of the metabolic enzymes, transporters, and GTs required for HMO biosynthesis and crucial enzymes like lactose synthase can be readily added as demonstrated. Additional genetic editing can potentially unlock the biosynthesis of more complex HMOs, an approach that remains technically challenging in microbial systems due to limited genetic engineering space (Zhang et al. 2019). Second, the human HEK293 cells can be grown in suspension cultures without animal-derived serum. Therefore, there is little risk of contamination with non-human factors during HMO purification, whereas purification from microbial cells needs to avoid endotoxin contamination (Bensimon J & Lu X, 2024; Lai et al. 2025). These advantages could make HEK293 cell-based HMO production an interesting alternative to microbial systems in future, but first scalability, recovery, and purification methods need to be developed.

In conclusion, we have developed a strategy for the selective production of lactose and 3’-SL, and 6’-SL in glycoengineered HEK293 cells. Further genetic engineering for expression of other GT combinations may allow expanding the HMO diversity that can be produced in this human cell-based system.

## Materials and methods

### Transcriptomic data analysis

Expression data for glycosyltransferases in HEK293 cells and human mammary epithelial cells were obtained from previously published transcriptomic datasets E-MTAB-9841 (Batch 1), E-MTAB-10855 (Batch 2), and E-MTAB-10885 (Narimatsu et al. 2019; Twigger et al. 2022). For HEK293 cells, gene expression levels quantified as fragments per kilobase of transcript per million mapped reads (FPKM) were extracted and plotted. For lactating mammary luminal cells, expression values correspond to log-transformed counts per million (logCPM) derived from differential expression analyses comparing lactating and non-lactating epithelial subpopulations. The presence or absence of transcripts encoding selected glycosyltransferases was determined based on detectable expression in these datasets with thresholds FPKM >1 applied for HEK293 cells and logCPM >1 for human mammary epithelial cells.

### Cell culture and transfection

Previously generated isogenic HEK293 cell lines (Bull et al. 2021) and HeLa cells were cultured in Dulbecco’s Modified Eagle Medium (DMEM) with high glucose and L-glutamine (Gibco) supplemented with 10% heat-inactivated FBS (Gibco) and 1× penicillin/streptomycin (Gibco) at 37 °C and 5% CO_2_ in a humidified incubator. Cells were passaged every 2–3 days and used until a maximum passage number of thirty after resuscitation from liquid nitrogen storage. pIRES-eGFP LALBA and pIRES-eGFP B4GALT1 were constructed by restriction-based ligation (NheI-HF/XhoI) of LALBA or B4GALT1 gene from pTWIST LALBA or pTWIST B4GALT1 (Twist Bioscience) into pIRES-EGFP-PURO (Addgene: #45567, Supplementary Fig. 23 and 24). Constructed plasmids were sequenced using the Forward primer: 5’-GTGTACGGTGGGAGGTC-3′ and Reverse primer 5’-TACCGTCGACTGCAGAA-3′ binding to the CMV promotor and IRES2 domain respectively. Transfections were performed using Lipofectamine 3000 (Invitrogen) according to the manufacturers’ protocol. For western blot and HMO analysis, HEK293 cells at a 60–80% confluency were co-transfected with total of 1.5 μg pIRES-eGFP plasmid DNA encoding either B4GALT1–6xHis or LALBA-FLAG (1:1 ratio) (Supplementary Fig. 23). For immunocytochemistry, HeLa cells were seeded 24 h prior to transfection onto φ 16 mm coverslips in 24-well plates coated with 1 mg/mL poly-L-lysine (Sigma-Aldrich) at a density of 0.05 × 10^6^ cells/well. The HeLa cells were transfected with 0.5 μg pTWIST LALBA and 0.5 μg pTWIST B4GALT1 plasmids (Supplementary Fig. 24). Transfections were performed in biological triplicates with one passage number in-between transfection rounds.

### Western blotting

Transfected HEK293 cells were washed with 1× PBS and lysed in cell lysis buffer (150 mM NaCl, 50 mM Tris–HCL pH 7.5, 5 mM EDTA, 0.1% SDS, 1% Triton X-100, 1× protease inhibitor cocktail mix) for 1 h on ice. The lysates were centrifuged (21.000 g, 4 °C, 10 min) and the supernatants was transferred into clean 1.5 ml tubes. Protein concentrations were determined using the Pierce BCA Protein Assay kit (Thermo Scientific) and equal amounts of lysates were loaded on 10% SDS-PAGE gels (for B4GALT1 detection) and 12% SDS-PAGE gels (for LALBA detection). Proteins were transferred onto PVDF membranes (Roche) using transfer conditions of 325 mA and 60 min for B4GALT1 detection and 325 mA and 30 min for LALBA detection. The membranes were blocked with 5% non-fat dry milk (NFDM) in PBS-T (0.05% Tween 20 in 1× PBS) for 1 h at room temperature (RT). Membrane were stained for 1 h at RT with mouse anti-6xHis AF647 (Biolegend) or mouse anti-FLAG AF647 (BioLegend) antibodies (1 μg/mL in PBS-T containing 1% NFDM). Membranes were washed trice with PBS-T for 5 min at RT and imaged using G:BOX F3 system (Syngene).

### Immunocytochemistry

At 48 h post-transfection, HeLa cells grown on coverslips were gently washed with 1× PBS and fixed with 4% paraformaldehyde for 10 min at RT. After three washing steps with 1× PBS, the cells were incubated for 10 min at RT with permeabilization buffer (1× PBS, 0.25% Triton X-100, 1% bovine serum albumin (BSA). After washing with PBS-T, the coverslips were blocked with 1× PBS with 5% BSA for 1 h at RT. Cells were then incubated for 1 h at RT with 1 μg/mL primary antibodies diluted in 1× PBS with 1% BSA. Mouse anti-6xHis (Invitrogen) and mouse anti-FLAG (Invitrogen) were used for detection of B4GALT1 and LALBA, respectively. Rabbit anti-GM130 (1 μg/mL; Invitrogen) was included as a Golgi marker. After washing trice with PBS-T, the coverslips were incubated for 1 h at RT with 1 μg/mL goat anti-mouse IgG AF488 (Invitrogen) and 1 μg/mL IRDYE 680RD goat anti-rabbit IgG (LICORbio) in 1× PBS with 1% BSA. After two washing steps with PBS-T and two washing steps with 1× PBS, coverslips were incubated with 0.5 μg/mL DAPI (Sigma-Aldrich) in 1× PBS for 5 min at RT, followed by washing once with 1× PBS and once with 0.1 M phosphate buffer (Na_2_HPO_4_/NaH_2_PO_4_, pH 7.4). Cover slips were mounted onto microscope slides using Dako fluorescent mounting medium (Agilent) and were imaged using an EVOS M3000 imaging system (Invitrogen) with 40× magnification.

### Sample preparation for UPLC-FD

At 72 h post-transfection, supernatants from HEK293 cell cultures were collected, centrifuged (21.000 g, 4 °C, 10 min), and transferred into clean 1.5 ml tubes. The wells were washed once with 1 mL 1× PBS and lysed in 1 mL Milli-Q water followed by five freeze–thaw cycles. The lysates were centrifuged (21.000 g, 4 °C, 10 min) and the supernatants were transferred into clean 1.5 ml tubes. Aliquots from supernatants or lysates (200 μL) were mixed with 10 μL Milli-Q water and 10 μL internal standard solution (500 mg/L laminaritriose, Megazyme). Proteins were precipitated via Carrez precipitation by addition of 10 μL Carrez solution 1 (Carl Roth) and 10 μL Carrez solution 2 (Carl Roth) followed by vortexing and centrifugation (16.000 g, 1 min, RT). After protein precipitation, 100 μL of the supernatant was taken and mixed with 100 μL label solution consisting of 34.67 mM 2-aminobenzamide (Sigma-Aldrich) and 1 M picoline borane (SigmαAldrich) in an acetic acid-DMSO mixture (3:7, v/v). Samples were incubated at 65 °C for 1 h under gentle shaking. Next, the samples were cooled down to RT followed by centrifugation (16.000 g, 1 min, RT) and addition of 1 mL 75% acetonitrile (LiChrosolv, VWR) in Milli-Q water. The samples were filtered over a 0.22 μm Millex PVDF syringe filter (Phenomenex) and 2 μL was injected on a Thermo Scientific Vanquish UPLC system equipped with a Waters Acquity BEH Premier Glycan column (130 Å, 1.7 μm, 2.1 × 150 mm) thermostated at 65 °C. Elution was performed at a flow rate of 0.5 mL/min using the following gradient of 50 mM ammonium formate pH 4.40 (Eluent A) and 100% ACN (Eluent B): 0–38 min linear gradient from 90 to 80.1% B, 38–38.5 min linear gradient from 80.1 to 20% B, 38.5–41.5 min isocratic on 20% B, 41.5–42 min linear gradient from 20 to 90% B, 42–52 min isocratic on 90% B. Elution was monitored using a Thermo Scientific Vanquish fluorescence detector with an emission and excitation wavelength of 260 and 420 nm, respectively. Each set of biological triplicates was measured over two days to ensure intermediate precision (one week in between analysis). One set was measured at all four days to ensure replication between days.

### Identification and quantification of lactose, 3’-SL, and 6’-SL

Lactose, 3’-SL, and 6’-SL were quantified using a calibration curve consisting of lactose monohydrate (Sigma-Aldrich), 3′-sialyllactose sodium salt (Carbosynth), and 6′-sialyllactose sodium salt (Carbosynth). To determine the recovery, one sample of each experimental condition was spiked with 10 μL spiking solution (47.6 mg/L lactose, 37 mg/L 3’-SL, and 37 mg/L 6’-SL) instead of 10 μL Milli-Q water. The samples were then prepared according to the general sample preparation for UPLC-FD.

### Hydrolysis of lactose, 3’-SL, and 6’-SL

For each lactose, 3’-SL, or 6’-SL positive condition, one sample was subjected to hydrolysis with β-galactosidase and neuraminidase. Aliqouts of supernatant or lysate (200 μL) were mixed with 8 μL Milli-Q water, 1 μL neuraminidase (100 mU, *Clostridium perfringens*, Roche), and 1 μL β-galactosidase (100 mU, *E. coli*, Sigma-Aldrich). Subsequently, the samples were incubated for 5 h at 37 °C followed by heat inactivation (65 °C, 10 min). The samples were then prepared according to the general sample preparation for UPLC-FD.

### Data visualization and statistical analysis

DNA sequencing files were aligned with template DNA encoding for LALBA or B4GALT1, using the Benchling Alignment Tool with the MAFFT (auto) algorithm (Benchling). Graphs, figures, and western blots were processed using Python 3, Microsoft PowerPoint (Microsoft Corporation), and Excel (Microsoft Corporation). Microscopy images were processed using Fiji (ImageJ) (Schindelin et al. 2012). Chromatographic raw data files were processed using the Chromeleon Chromatography Data System (Thermo Fisher Scientific). Each peak was integrated manually via the valley-to-valley integration method, yielding the raw area under the curve value (AUC) and the retention time (RT). Values under the limit of quantifications were set to LOQ (LOQ_Lactose_ = 0.52 mg/L, LOQ_3’-SL_ and LOQ_6’-SL_ = 0.14 mg/L). Data are presented as mean ± standard deviation of the biological triplicates unless stated differently.

## Supplementary Material

Supplementary_material_cwag048

## Data Availability

All data presented in the figures and tables, and supplementary information of this paper are available.
