# Supplementary material for: Biosynthesis of human milk oligosaccharides (HMOs) in glycoengineered human cells
Source: Glycobiology. 2026 Jun 22;36(8):cwag048. doi: 10.1093/glycob/cwag048 (PMC13310140; doi:10.1093/glycob/cwag048)
Supplement: Supplementary_material_cwag048 [file supplementary_material_cwag048.zip › Supplementary_Information_Final-Revised_cwag048.docx]

**SUPPLEMENTARY INFORMATION**

**Biosynthesis of human milk oligosaccharides (HMOs) in glycoengineered human cells**

Stijn Kruf^1^, Roy J.B.M. Delahaije^2^, Khadra A. Mohamed^1^, Barry Schoemaker^2^, Yoshiki Narimatsu^3,4^, Henrik Clausen^3^, Vassilis Triantis^2^, Thomas J. Boltje^5,^*, Christian Büll^1,3^*

^1^ Department of Biomolecular Chemistry, Institute for Molecules and Materials, Radboud University, 6525 AJ, Nijmegen, the Netherlands

^2^ FrieslandCampina, Amersfoort, Netherlands

^3^ Copenhagen Center for Glycomics, Departments of Cellular and Molecular Medicine, Faculty of

Health Sciences, University of Copenhagen, Blegdamsvej 3, Copenhagen, Denmark

^4^ GlycoDisplay ApS, Copenhagen, Denmark

^5^ Synthetic Organic Chemistry, Institute for Molecules and Materials, Radboud University Nijmegen, Heyendaalseweg 135, 6525 AJ Nijmegen, The Netherlands

*Corresponding authors: *thomas.boltje@ru.nl* & *christian.bull@ru.nl*

**Table of content**

**Supplementary Table 1.** Sources and purity of HMO standards

**Supplementary Fig. 1.** Chromatograms of Milli-Q controls

**Supplementary Fig. 2.** Chromatogram of mock-transfected HEK239^WT^ supernatant

**Supplementary Fig. 3.** Western blot of LALBA/B4GALT1 expression in HEK293^WT^ and

HEK293^ΔSia^

**Supplementary Fig. 4.** Screening of 17 human milk oligosaccharides in supernatant of

LALBA/B4GALT1-transfected HEK293^WT^

**Supplementary Fig. 5.** Chromatograms of mock-transfected and LALBA/B4GALT1-transfected

HEK293WT supernatants

**Supplementary Fig. 6.** Chromatograms of LALBA/B4GALT1-transfected HEK293^WT^ supernatant

spiked with lactose, 3’-sialyllactose, and 6’-sialyllactose

**Supplementary Fig. 7.** Chromatograms of LALBA/B4GALT1-transfected HEK293^WT^ supernatant

before and after enzymatic hydrolysis

**Supplementary Fig. 8.** Chromatograms of mock-transfected and LALBA/B4GALT1-transfected

HEK293^ΔSia^ supernatants

**Supplementary Fig. 9.** Chromatograms of LALBA/B4GALT1-transfected HEK293^ΔSia^ supernatant

spiked with lactose, 3’-sialyllactose, and 6’-sialyllactose

**Supplementary Fig. 10.** Chromatograms of LALBA/B4GALT1-transfected HEK293^ΔSia^ supernatant

before and after enzymatic hydrolysis

**Supplementary Fig. 11.**  Recovery of lactose, 3’-sialyllactose, and 6’-sialyllactose in supernatant of

HEK293 cell lines

**Supplementary Fig. 12.** Recovery of lactose, 3’-sialyllactose, and 6’-sialyllactose in HEK293 lysates

**Supplementary Fig. 13.** Inter-day variation of lactose and 3’-sialyllactose in HEK293^WT^ supernatant

**Supplementary Fig. 14.** Chromatograms lysates from mock-transfected and LALBA/B4GALT1-

transfected HEK293^WT^ cells

**Supplementary Fig. 15.** Western blot of LALBA/B4GALT1 expression in HEK293^KI ST3GAL1-6^

**Supplementary Fig. 16.** Chromatograms of mock-transfected and LALBA/B4GALT1-transfected

HEK293^KI ST3GAL5^ supernatants

**Supplementary Fig. 17.** Chromatograms of LALBA/B4GALT1-transfected HEK293^KI ST3GAL5^

supernatant spiked with lactose, 3’-sialyllactose, and 6’-sialyllactose

**Supplementary Fig. 18.** Chromatograms of LALBA/B4GALT1-transfected HEK293^KI ST3GAL5^

supernatant before and after enzymatic hydrolysis

**Supplementary Fig. 19.** Western blot of LALBA/B4GALT1 expression in HEK293^KI ST6GAL1/2^

**Supplementary Fig. 20.** Chromatograms of mock-transfected and LALBA/B4GALT1-transfected

HEK293^KI ST6GAL2^ supernatants

**Supplementary Fig. 21.** Chromatograms of LALBA/B4GALT1-transfected HEK293^KI ST6GAL2^

supernatant spiked with lactose, 3’-sialyllactose, and 6’-sialyllactose

**Supplementary Fig. 22.** Chromatograms of LALBA/B4GALT1-transfected HEK293^KI ST6GAL2^

supernatant before and after enzymatic hydrolysis

**Supplementary Fig. 23.** Schematic depiction of pIRES-eGFP plasmids

**Supplementary Fig. 24.** Schematic depiction of pTWIST plasmids.

**Supplementary Table 1.** Sources and purity of standards used for the screening of 17 human milk oligosaccharides in supernatant of LALBA/B4GALT1-transfected HEK293^WT^.


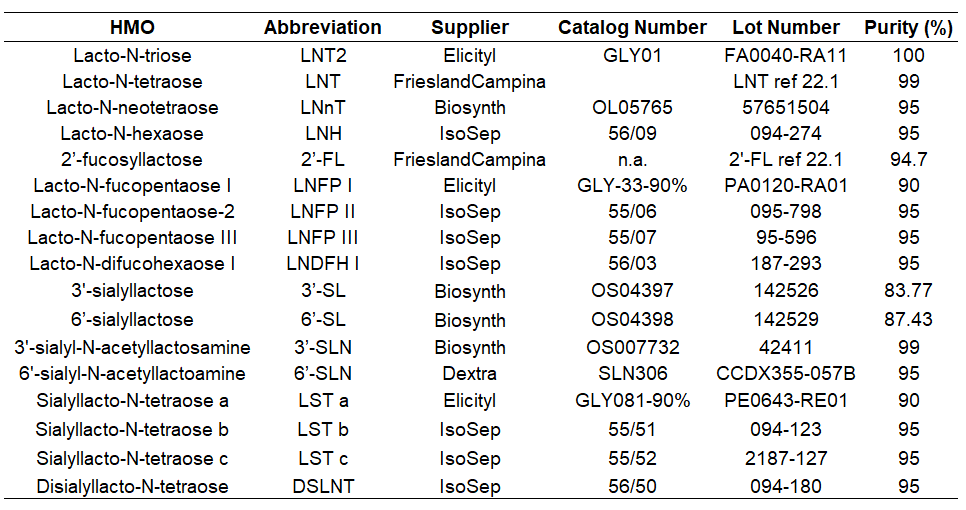


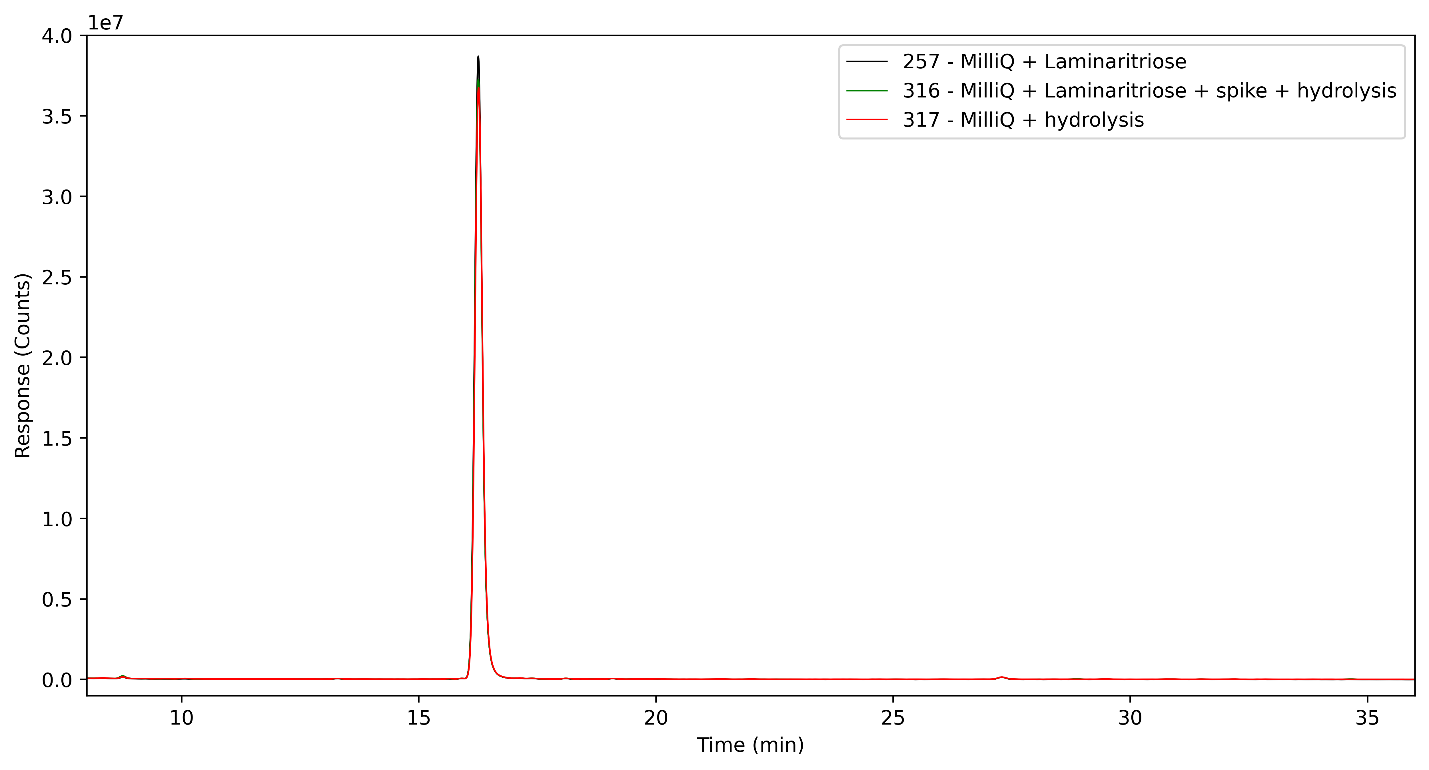
**Supplementary Fig. 1.** Chromatograms of Milli-Q controls. Overlay of representative chromatograms derived from UPLC-FD analysis of milliQ water with internal standard laminaritriose (black line), Milli-Q water after galactosidase and neuraminidase treatment (red line), and milliQ water with laminaritriose spiked with lactose, 3’-SL and 6’-SL after galactosidase and neuraminidase treatment (green line).


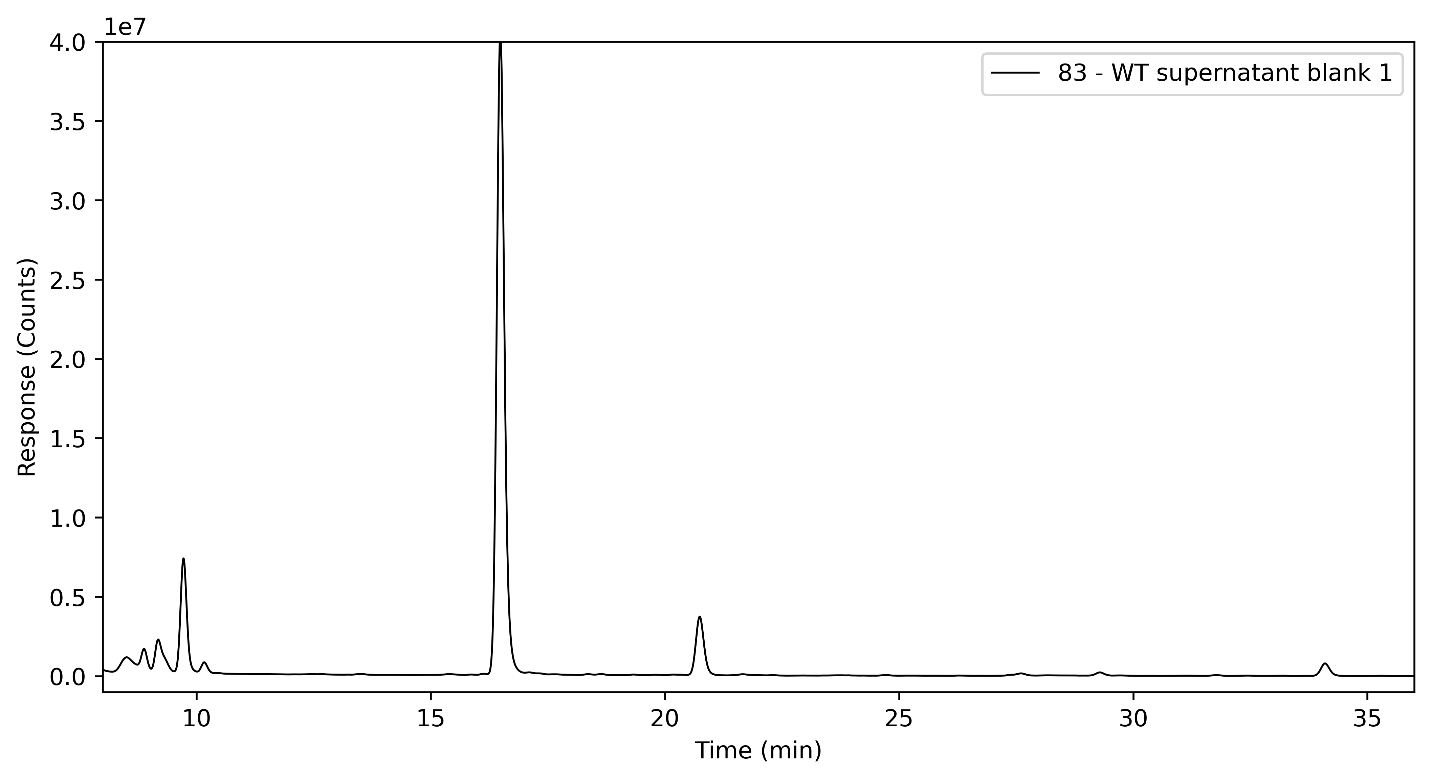
**Supplementary Fig. 2.** Chromatogram of mock-transfected HEK239^WT^ supernatant. Representative UPLC-FD chromatogram of supernatant derived from HEK293^WT^ cultures 72h after mock-transfection. Major peak corresponds to the internal laminaritriose standard and smaller peaks represent reducing carbohydrates in the culture supernatant.


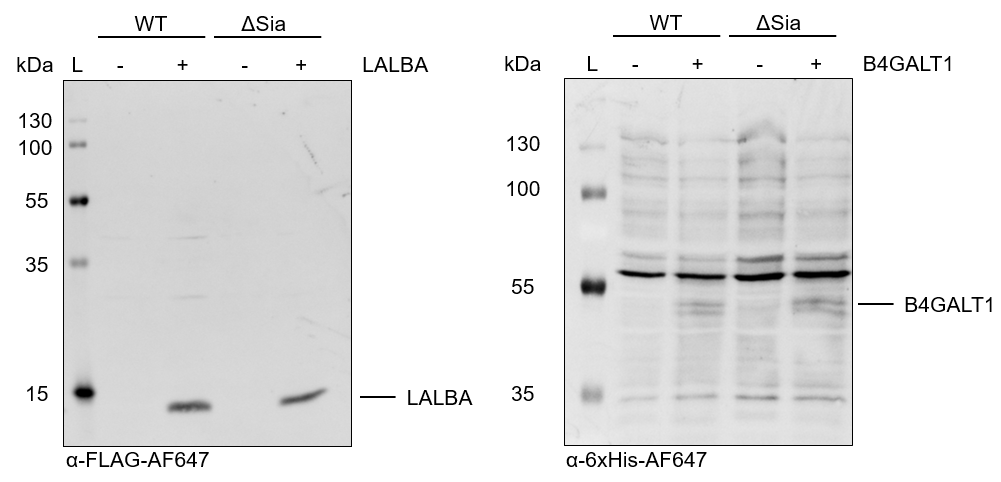
**Supplementary Fig. 3.** Western blot of LALBA and B4GALT1 expression in HEK293^WT^ and HEK293^ΔSia^. Representative western blots show expression of LALBA and B4GALT1 in HEK293^WT^ and HEK293^ΔSia^ cells transfected with pIRES-eGFP LALBA and pIRES-eGFP B4GALT1 plasmids. At 72h post-transfection, cells were lysed and samples were separated using a 12% (left) or 10% (right) SDS-PAGE gel following transfer onto a PVDF membrane. Membranes were stained with AF647-conjugated mouse anti-FLAG (left) or mouse anti-6xHis (right) antibodies, respectively.

**
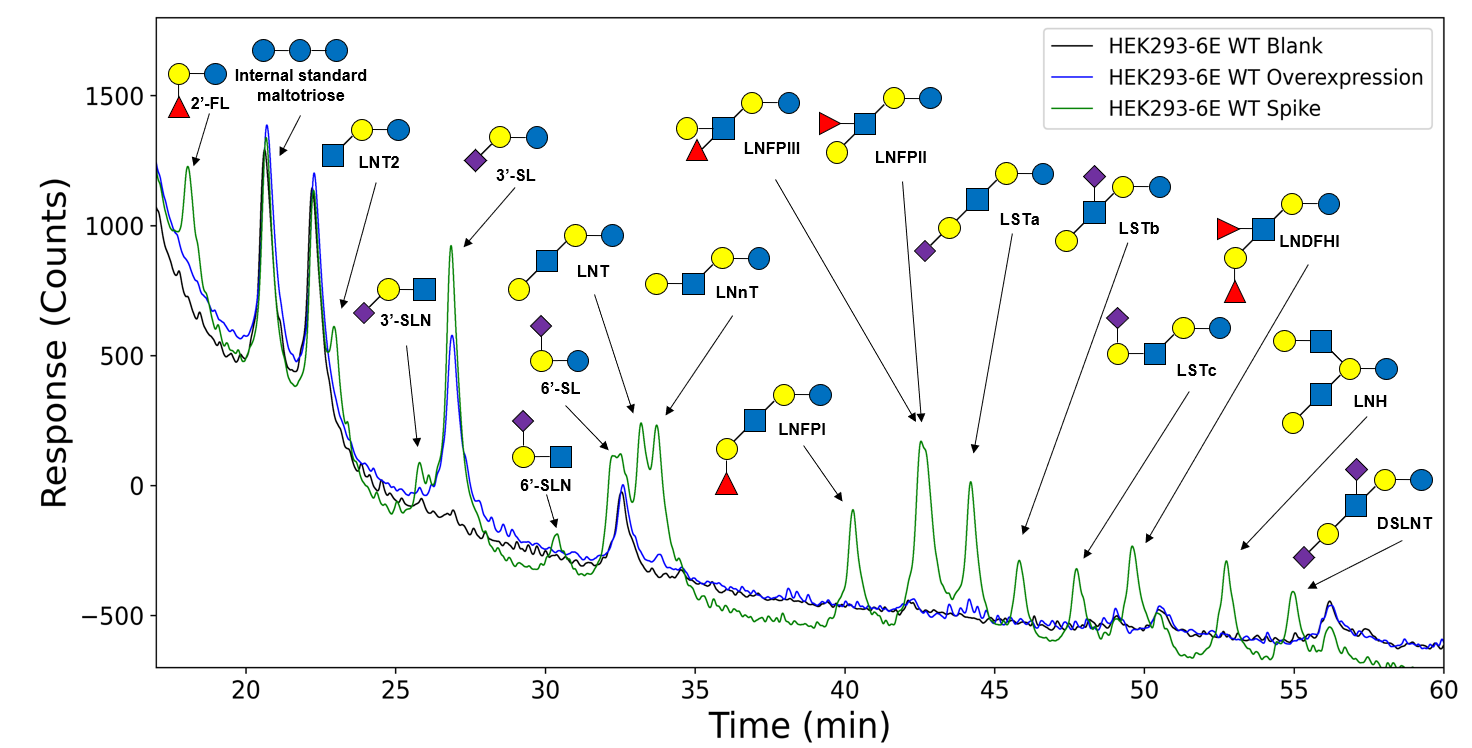
Supplementary Fig. 4.** Screening of 17 human milk oligosaccharides in supernatant of LALBA/B4GALT1-transfected HEK293^WT^. Overlay of representative UPLC-FD chromatograms of supernatants collected form mock-transfected HEK293^WT^ cells (black line), HEK293^WT^ with LALBA/B4GALT1 overexpression (blue line), and mock-transfected HEK293^WT^ supernatant with spike solution (green line). The spike solution contained a mixture of human milk oligosaccharides; 2-fucosyllactose (2’-FL),, lacto-N-triaose II (LNT2), 3'-sialyl-N-acetyllactosamine (3’-SLN), 3’-sialyllactose (3’-SL), 6'-sialyl-N-acetyllactosamine (6’-SLN), 6’-sialyllactose (6’-SL), lacto-N-tetraose (LNT), lacto-N-neotetraoese (LNnT), lacto-N-fucopentaose I (LNFPI), lacto-N-fucopentaose II (LNFPII), lacto-N-fucopentaose III (LNFPIII), siallyllacto-tetrasaccharide a (LSTa), siallyllacto-tetrasaccharide b (LSTb), siallyllacto-tetrasaccharide c (LSTc), lacto-N-difucohexaose I (LNDFHI), lacto-N-hexaose (LNH), and disialyllacto-N-tetraose (DSLNT), and maltotriose as internal standard, eluting from 17 to 60 minutes, respectively.


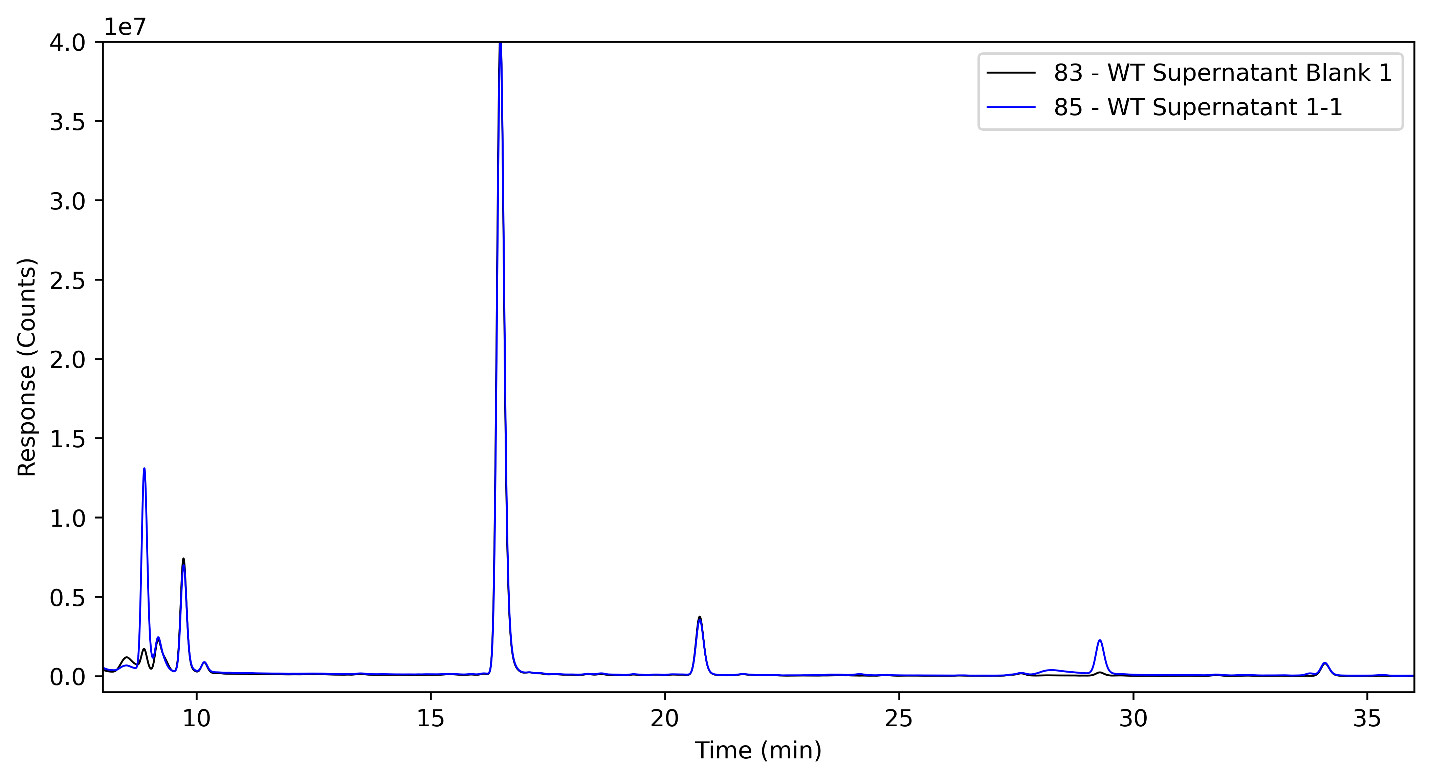
**Supplementary Fig. 5.** Chromatograms of mock-transfected and LALBA/B4GALT1-transfected HEK293^WT^ supernatant. Overlay of representative UPLC-FD chromatograms of mock-transfected HEK293^WT^ supernatant(black line) and LALBA/B4GALT1-transfected HEK293^WT^ supernatant (blue line). Retention time 8.8 min corresponds to lactose and 29.3 min to 3’-SL.

**Supplementary Fig. 6.** Chromatograms of LALBA/B4GALT1-transfected HEK293^WT^ supernatant spiked with lactose, 3’-sialyllactose, and 6’-sialyllactose. Overlay of representative UPLC-FD chromatograms for LALBA/B4GALT1-transfected HEK293^WT^ supernatant (blue line) and LALBA/B4GALT1-transfected HEK293^WT^
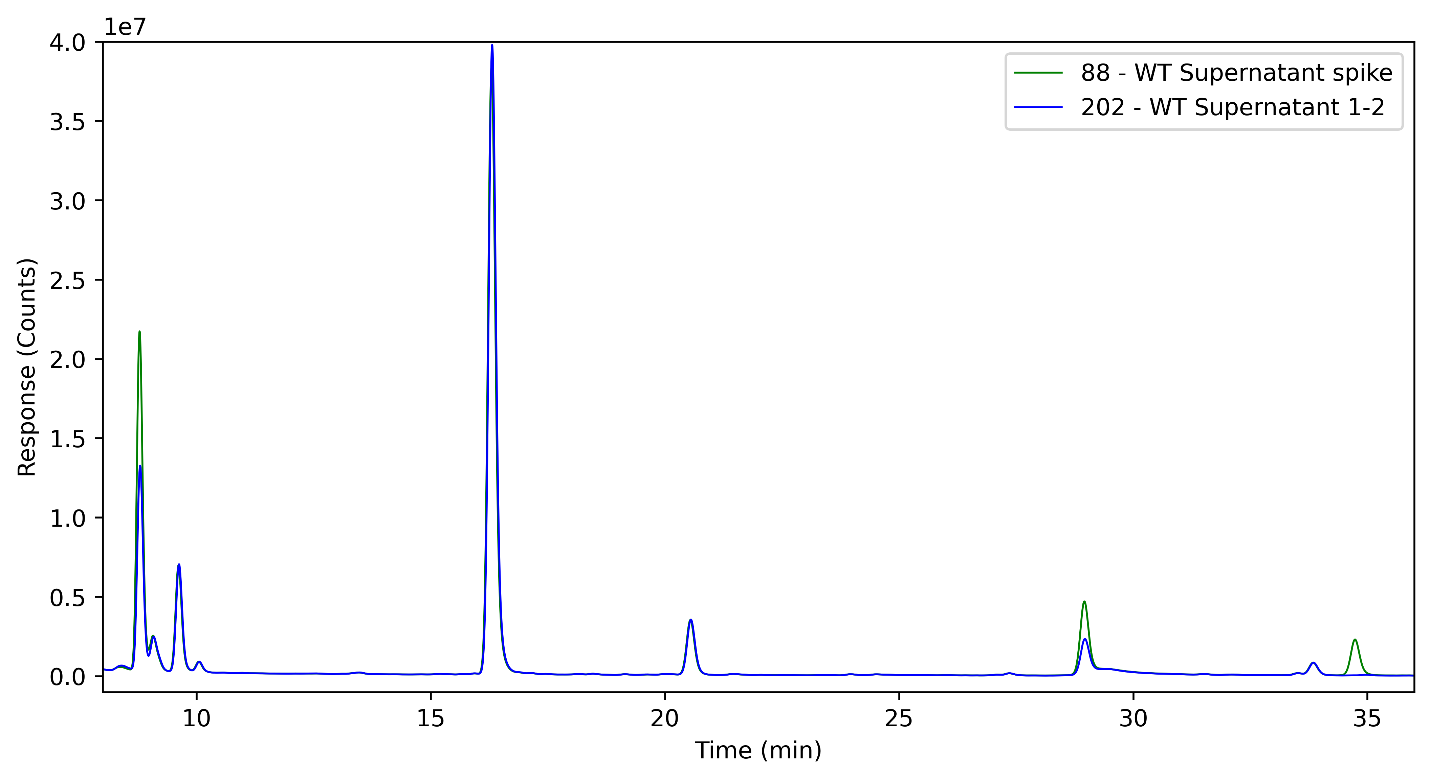
supernatant spiked with lactose, 3’-SL, and 6’-SL (green line). Retention time 8.8 min corresponds to lactose and 29.3 min to 3’-SL.


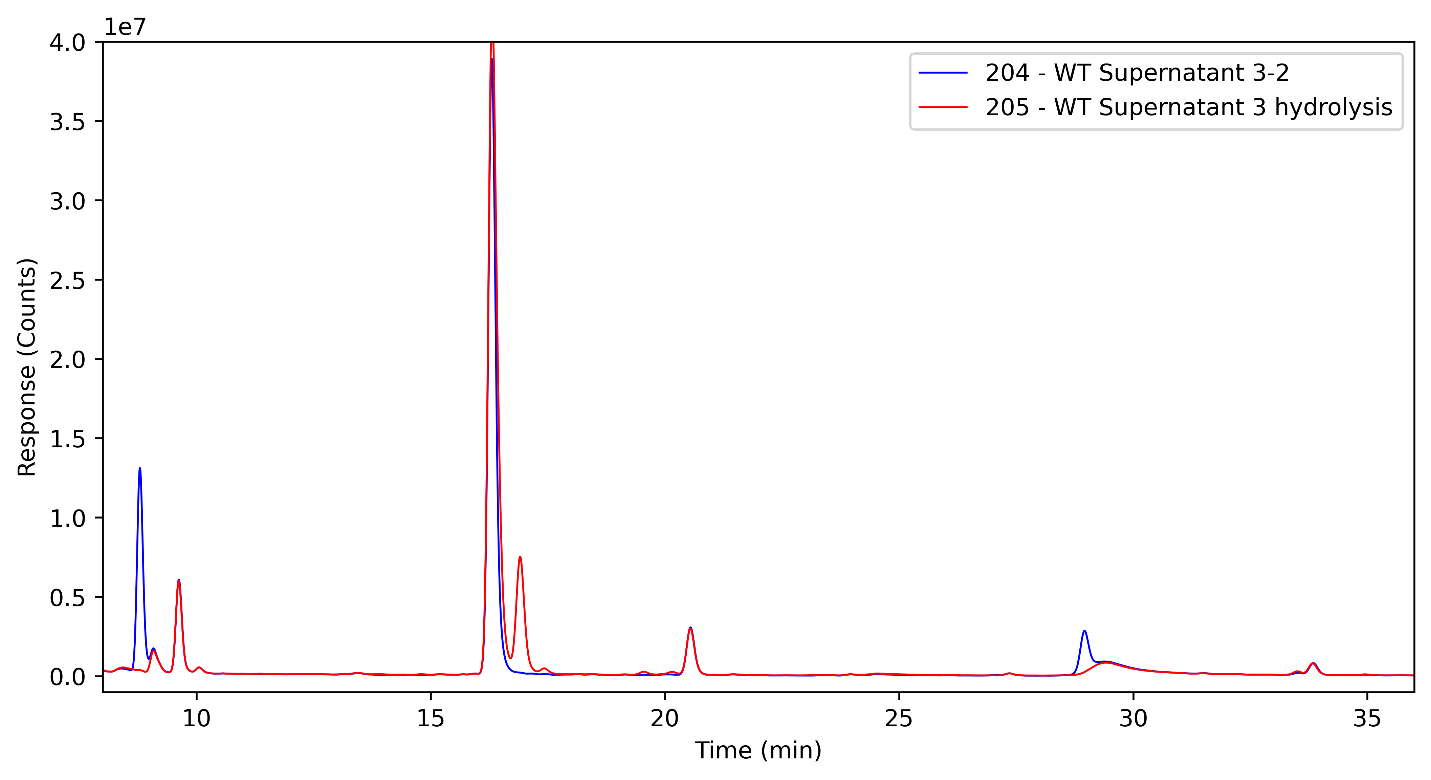
**Supplementary Fig. 7.** Chromatograms of LALBA/B4GALT1-transfected HEK293^WT^ supernatant before and after enzymatic hydrolysis. Overlay of representative UPLC-FD chromatograms of LALBA/B4GALT1-transfected HEK293^WT^ supernatant (blue line) and LALBA/B4GALT1-transfected HEK293^WT^ supernatant after galactosidase and neuraminidase treatment (red line). Retention time 8.8 min corresponds to lactose and 29.3 min to 3’-SL.

**
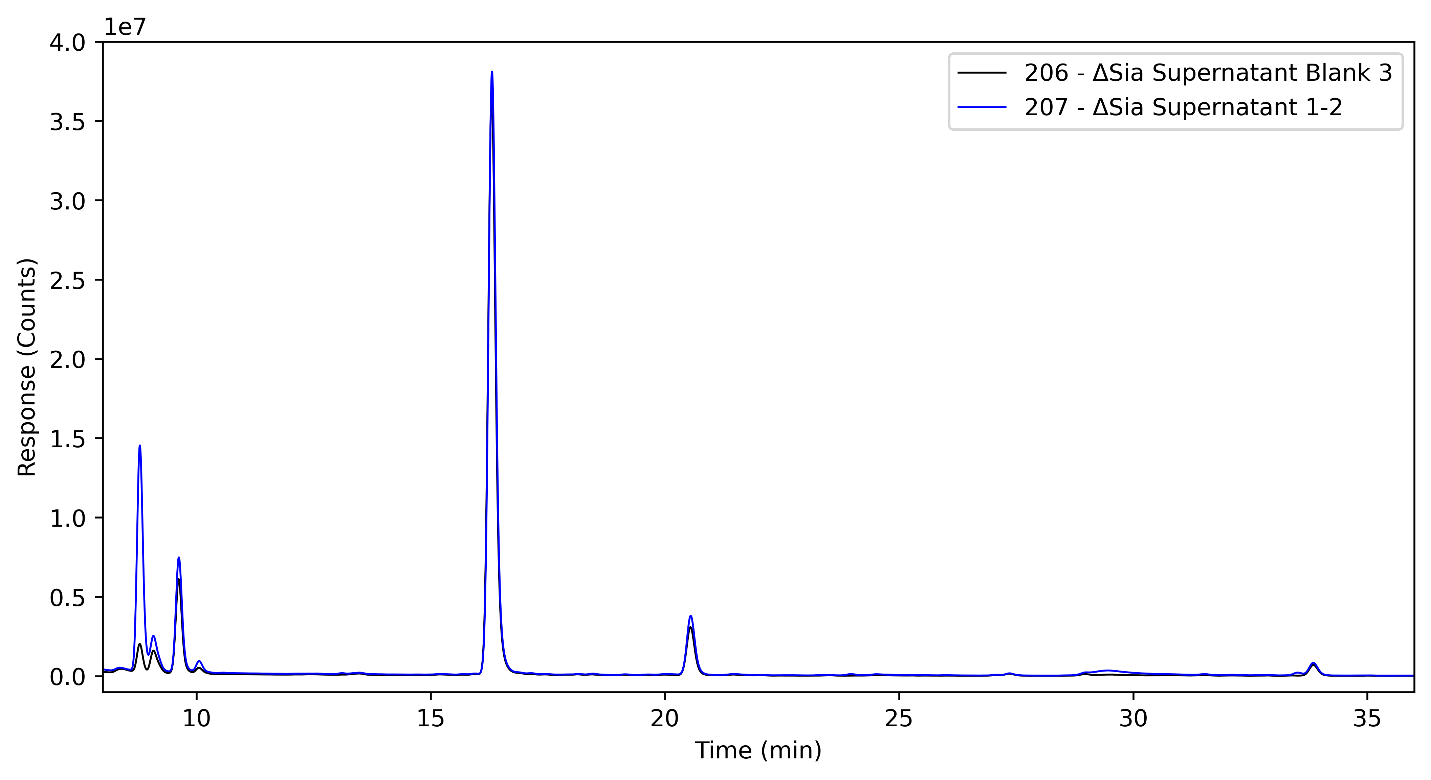
Supplementary Fig. 8.** Chromatograms of mock-transfected and LALBA/B4GALT1-transfected HEK293^ΔSia^ supernatant. Overlay of representative UPLC-FD chromatograms of mock-transfected HEK293^ΔSia^ supernatant (black line) and LALBA/B4GALT1-transfected HEK293^ΔSia^ supernatant (blue line). Retention time 8.8 min corresponds to lactose and 29.3 min to 3’-SL.


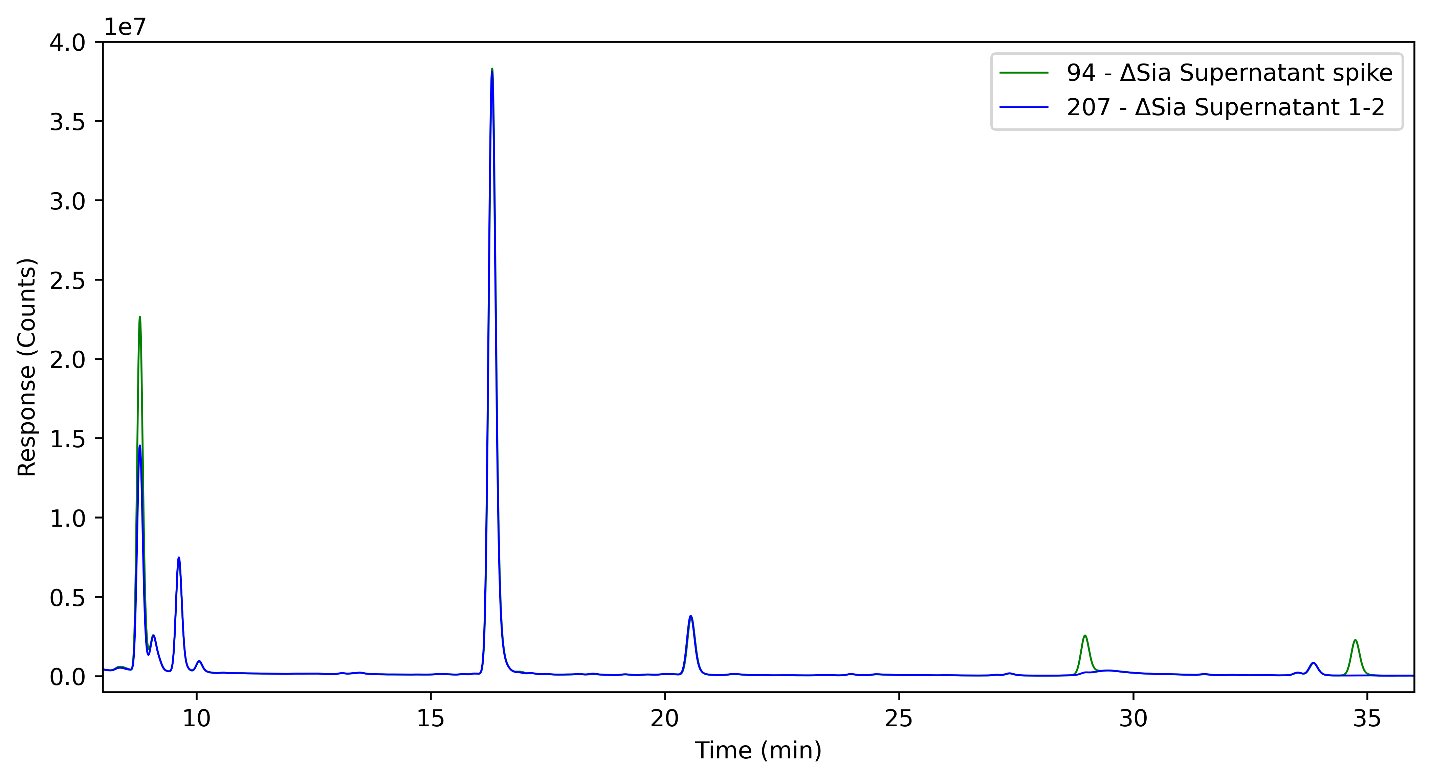


**Supplementary Fig. 9.** Chromatograms of LALBA/B4GALT1-transfected HEK293^ΔSia^ supernatant spiked with lactose, 3’-sialyllactose, and 6’-sialyllactose. Overlay of representative UPLC-FD chromatograms of LALBA/B4GALT1-transfected HEK293^ΔSia^ supernatant (blue line) and LALBA/B4GALT1-transfected HEK293^ΔSia^ supernatant spiked with lactose, 3’-SL and 6’-SL (green line). Retention time 8.8 min corresponds to lactose and 29.3 min to 3’-SL.


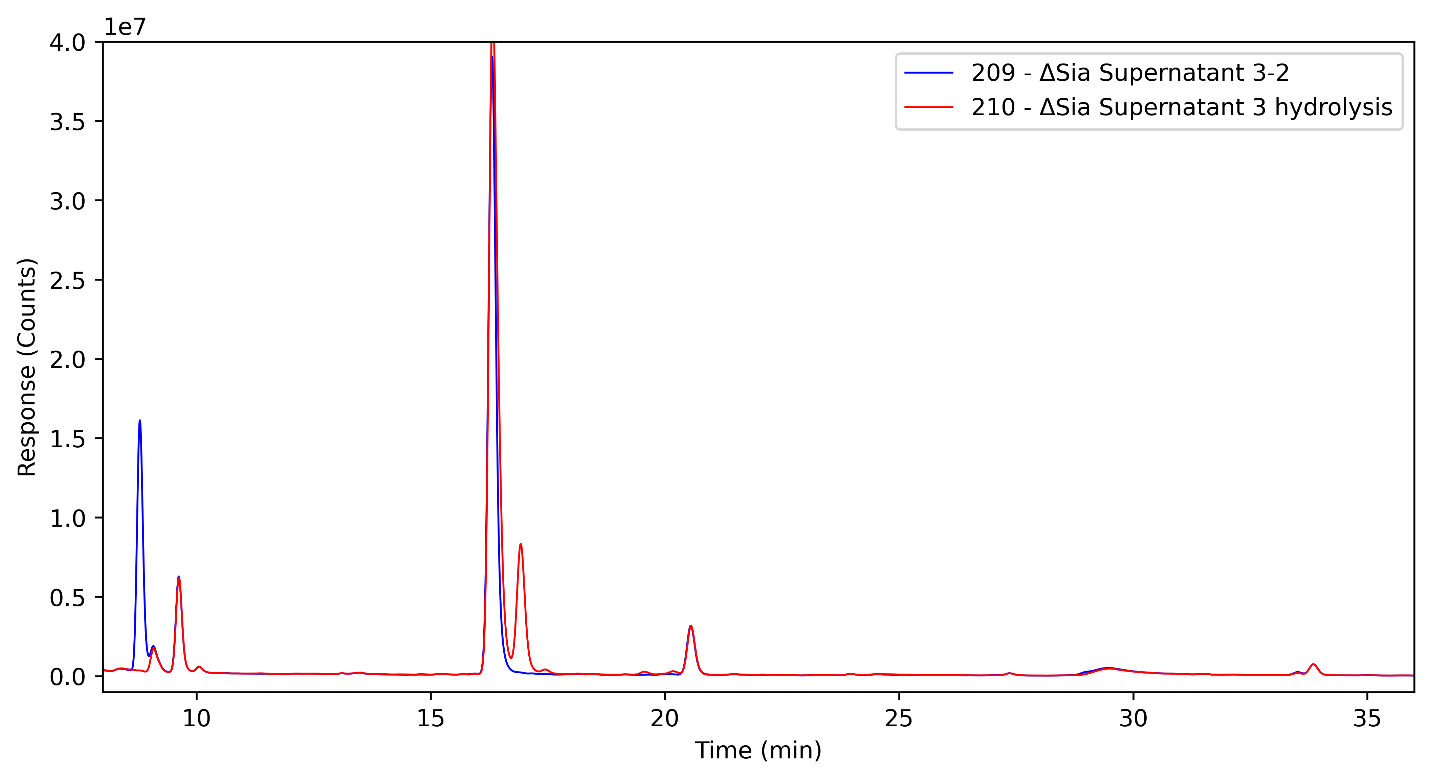
**Supplementary Fig. 10**. Chromatograms of LALBA/B4GALT1-transfected HEK293^ΔSia^ supernatant before and after enzymatic hydrolysis. Overlay of representative UPLC-FD chromatograms of LALBA/B4GALT1-transfected HEK293^ΔSia^ supernatant (blue line) and LALBA/B4GALT1-transfected HEK293^ΔSia^ supernatant after galactosidase and neuraminidase treatment (red line). Retention time 8.8 min corresponds to lactose and 29.3 min to 3’-SL.


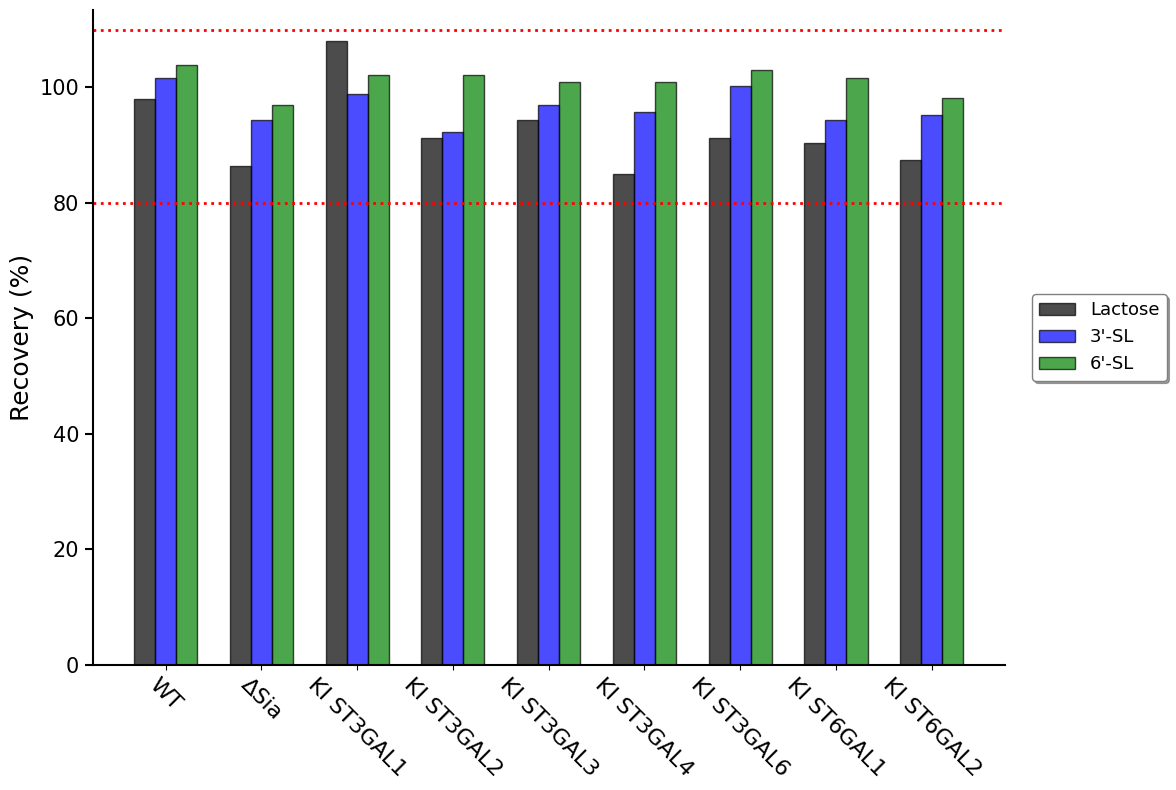
**Supplementary Fig. 11.** Recovery of lactose, 3’-sialyllactose, and 6’-sialyllactose in supernatants of HEK293 cell lines. Recovery of lactose, 3’-SL, and 6’-SL determined in LALBA/B4GALT1-transfected HEK293 supernatants after spiking with lactose, 3’-SL, and 6’-SL.

**
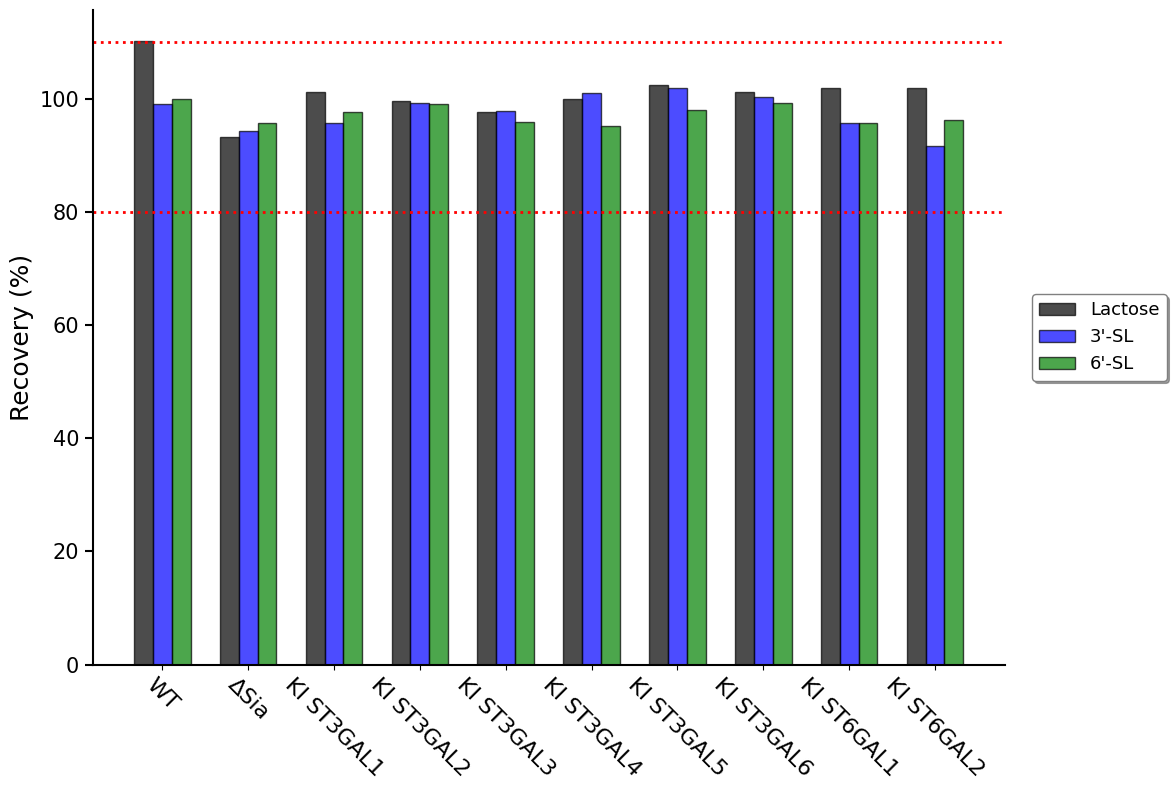
**

**Supplementary Fig. 12.** Recovery of lactose, 3’-sialyllactose, and 6’-sialyllactose in HEK293 lysates. Recovery of lactose, 3’-SL, and 6’-SL determined in LALBA/B4GALT1-transfected HEK293 lysates after spiking with lactose, 3’-SL, and 6’-SL.

**
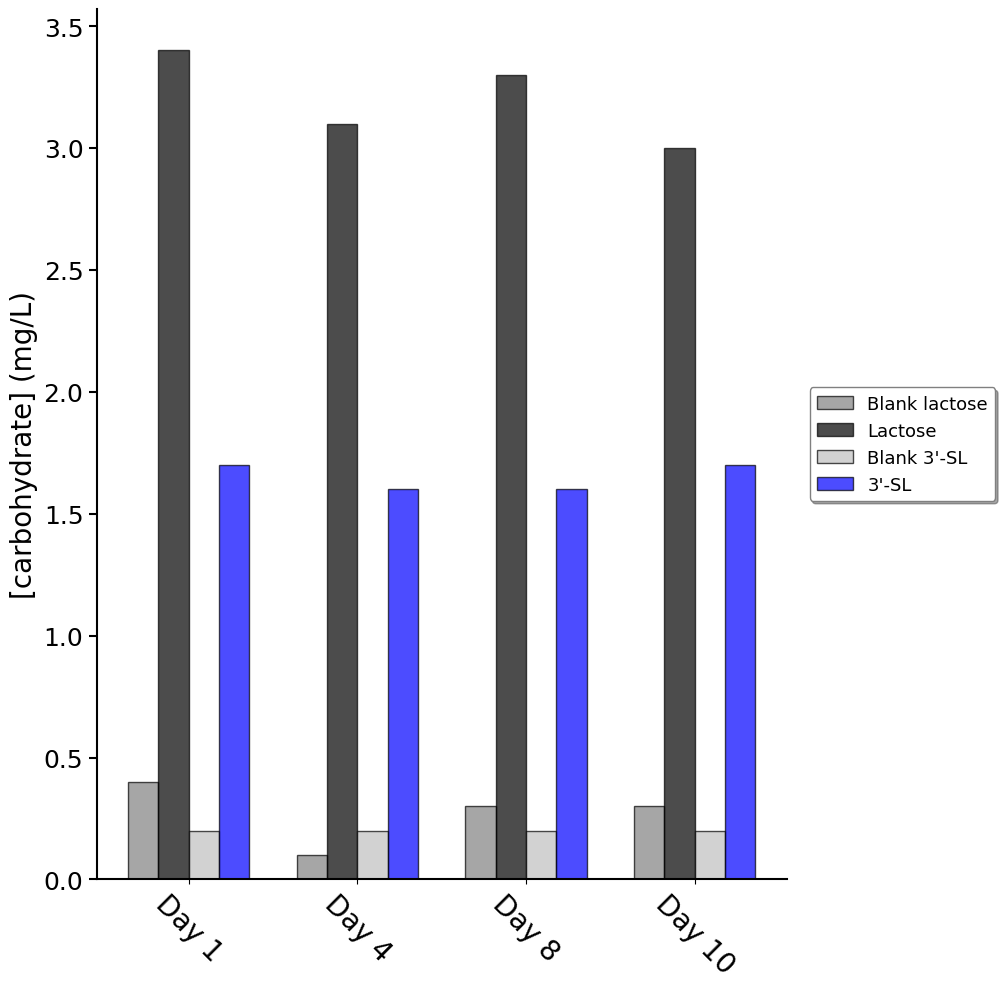
Supplementary Fig. 13.** Inter-day variation of lactose and 3’-sialyllactose in HEK293^WT^ supernatant. Lactose and 3’-SL content for mock-transfected HEK293^WT^ supernatant and LALBA/B4GALT1-transfected HEK293^WT^ supernatant in the same supernatant sample measured on different days by UPLC-FD. Relative standard deviation for inter-day variation was 5.5% and 3.8%, respectively.


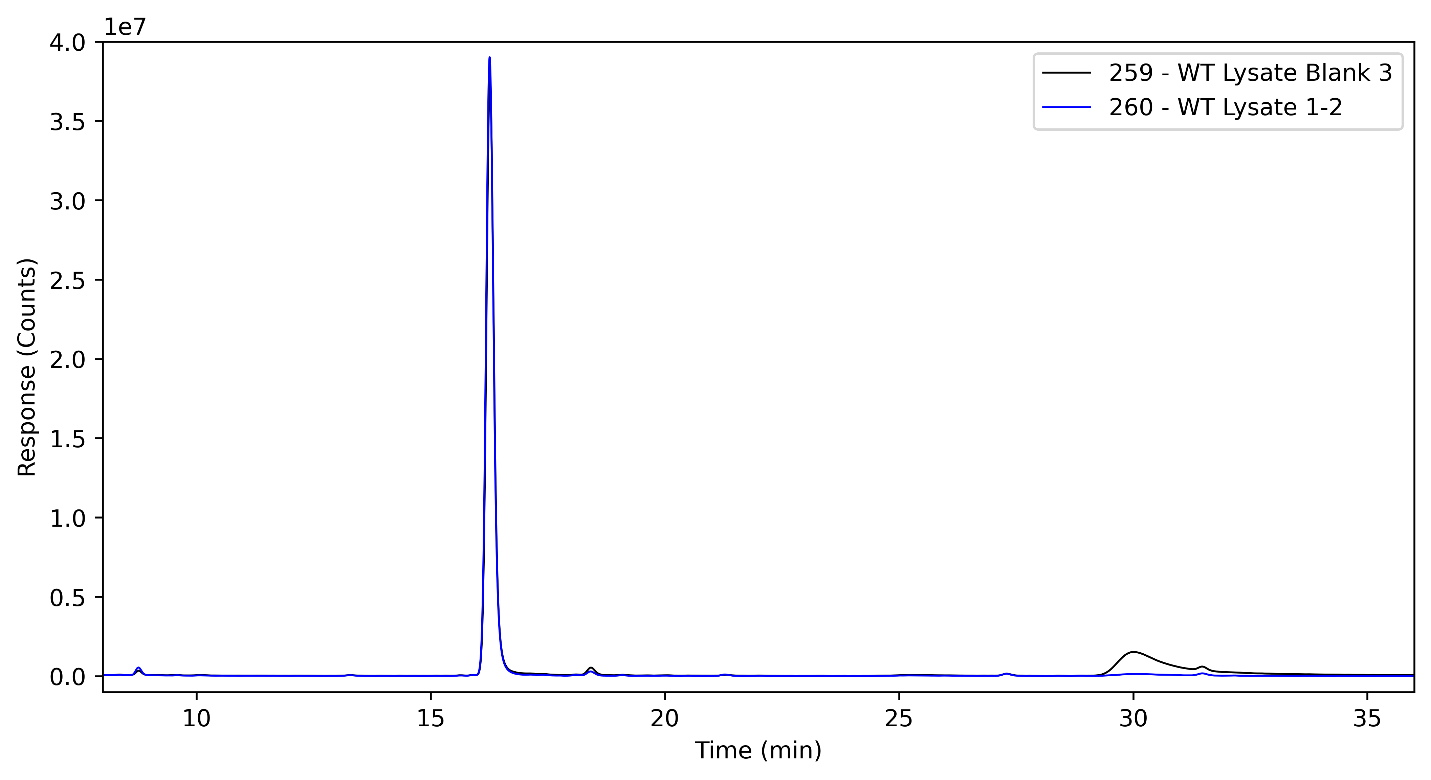


**Supplementary Fig. 14.** Chromatogram of lysates from mock-transfected and LALBA/B4GALT1-transfected HEK293^WT^ cells. Overlay of representative UPLC-FD chromatograms of mock-transfected HEK293^WT^ lysate (black line) and LALBA/B4GALT1-transfected HEK293^WT^ lysate (blue line).


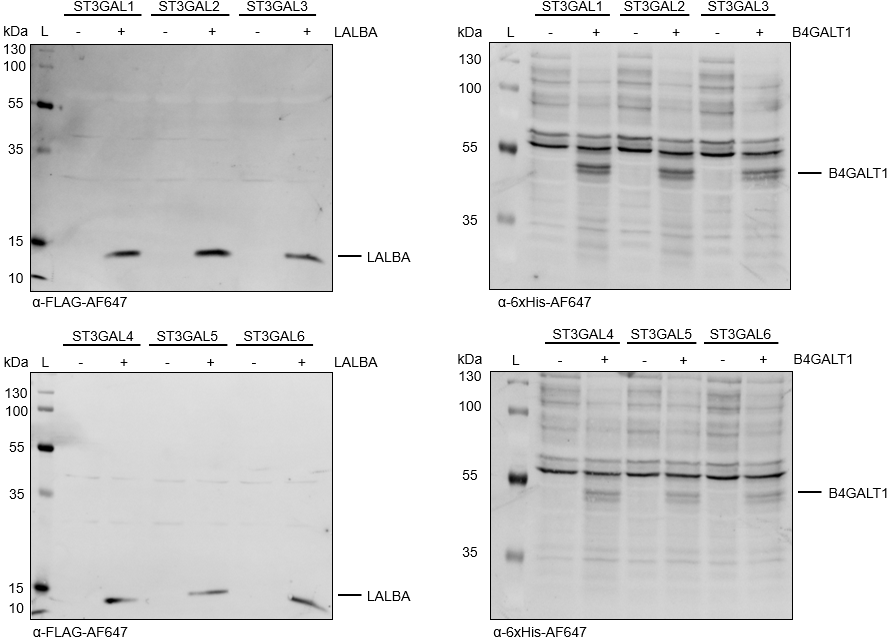
**Supplementary Fig. 15.** Western blot of LALBA/B4GALT1 expression in HEK293^ST3GAL1-6^. Cell lysates from HEK293^KI ST3GAL1-6^ cell lines co-transfected with FLAG-tagged LALBA and 6xHis-tagged B4GALT1 were subjected to SDS-PAGE/western blot analysis 72 h post-transfection. Membranes were stained with mouse α-FLAG-AF647 antibody (left) and mouse α-6xHis-AF647 antibody (right), respectively.

**
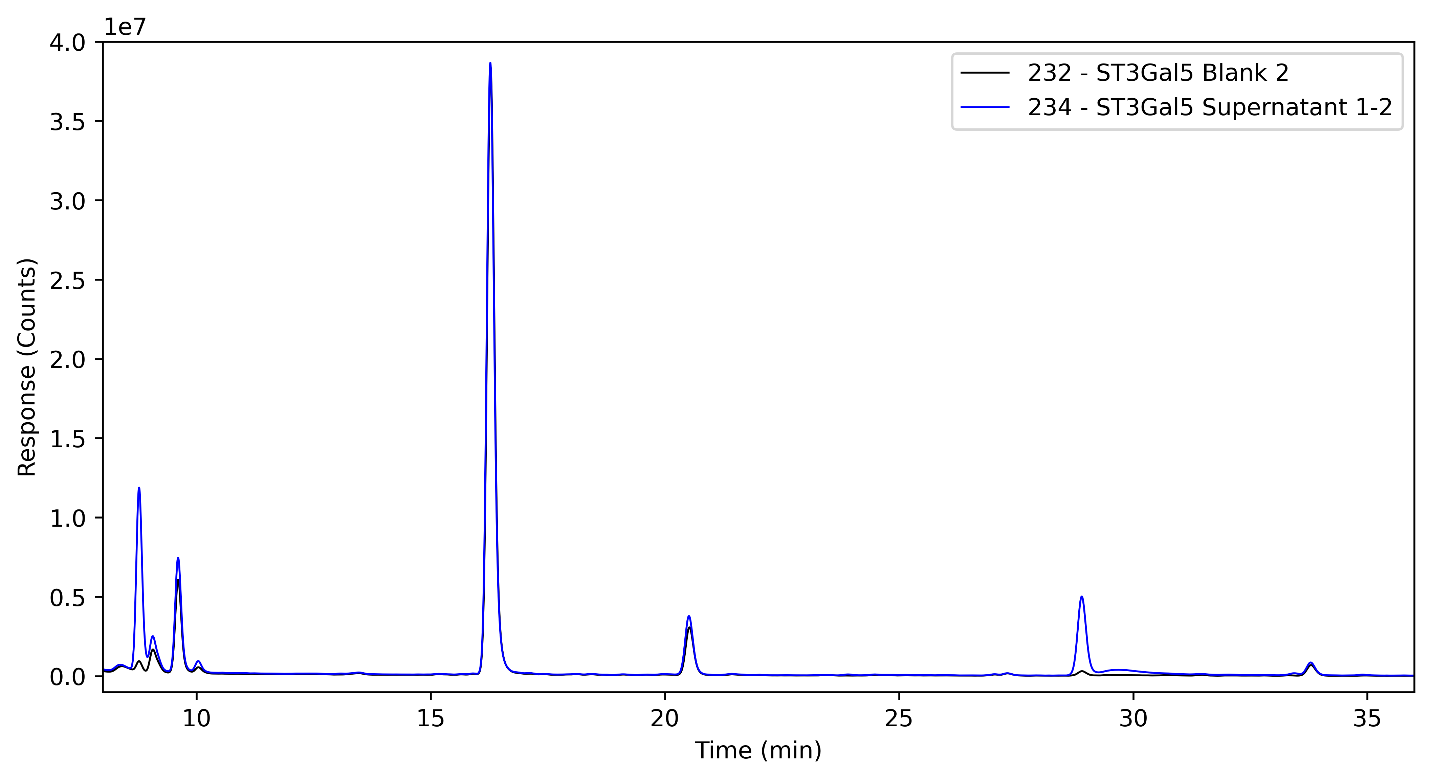
Supplementary Fig. 16.** Chromatograms of mock-transfected and LALBA/B4GALT1-transfected HEK293^ST3GAL5^ supernatant. Overlay of representative UPLC-FD chromatograms of HEK293^KI ST3GAL5^ supernatant blank (black line) and HEK293^KI ST3GAL5^ supernatant (blue line). Retention time 8.8 min corresponds to lactose and 29.3 min to 3’-SL.

**
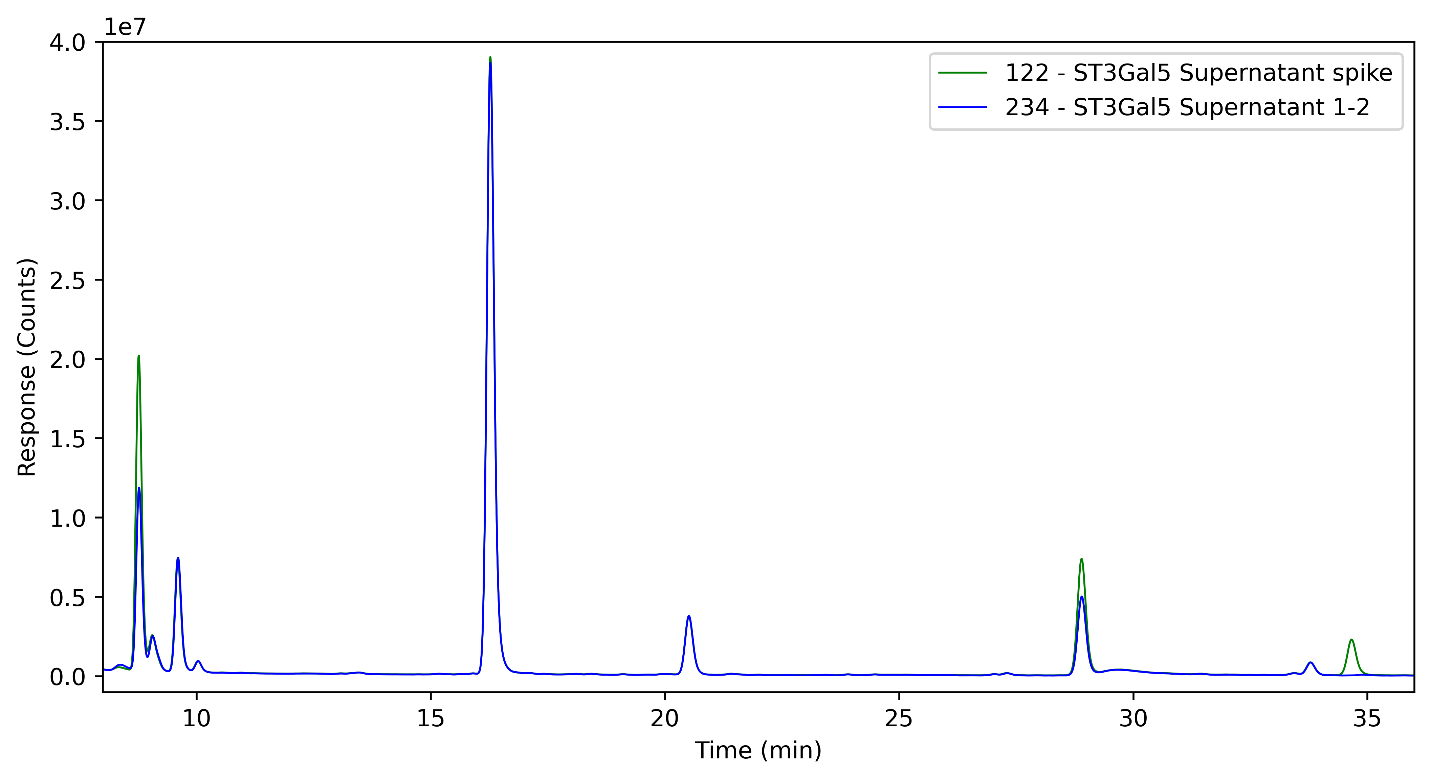
Supplementary Fig. 17.** Chromatograms of LALBA/B4GALT1-transfected HEK293^ST3GAL5^ supernatant spiked with lactose, 3’-sialyllactose, and 6’-sialyllactose. Overlay of representative UPLC-FD chromatograms of HEK293^KI ST3GAL5^ supernatant (blue line ) and HEK293^KI ST3GAL5^ supernatant spiked with lactose, 3’-SL and 6’-SL (green line). Retention time 8.8 min corresponds to lactose and 29.3 min to 3’-SL.

**
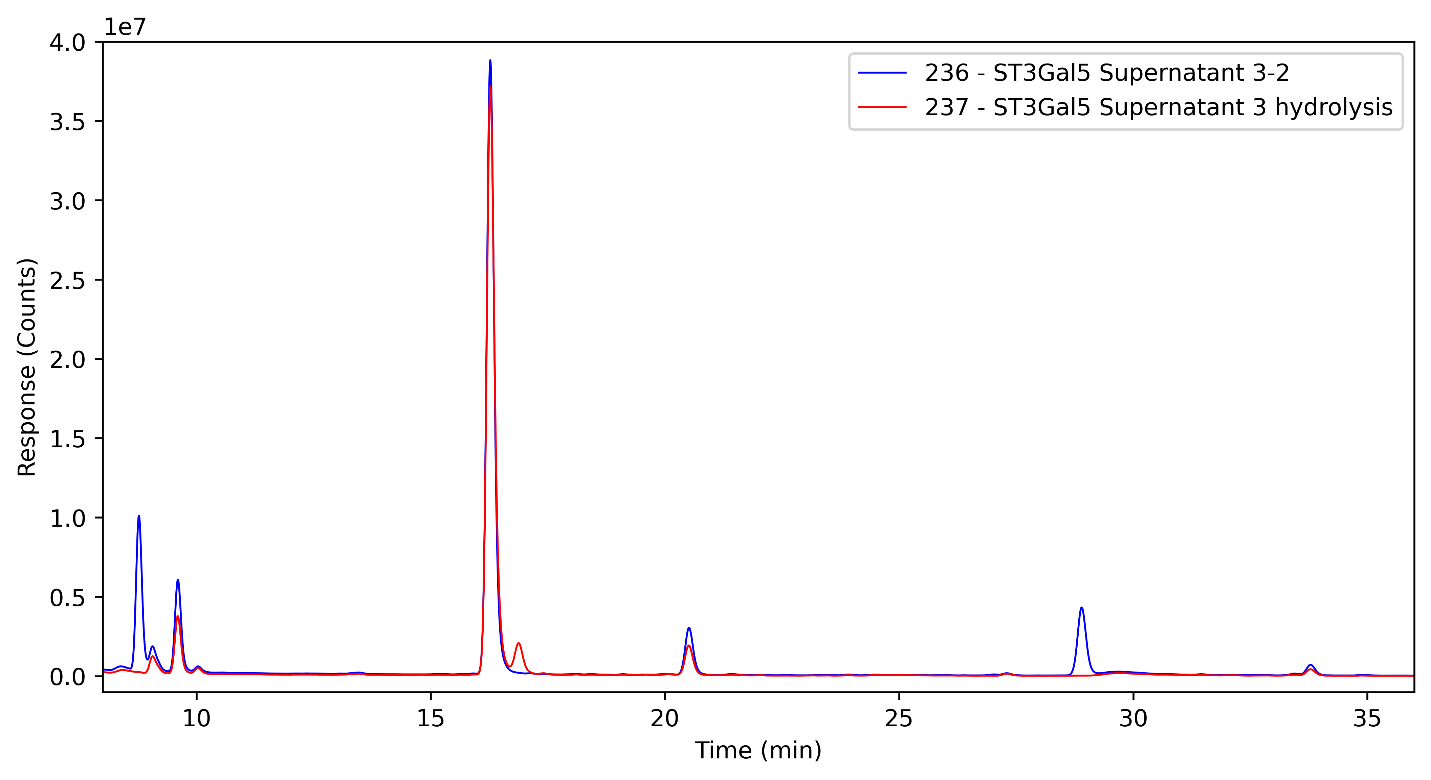
Supplementary Fig. 18**. Chromatograms of LALBA/B4GALT1-transfected HEK293^ST3GAL5^ supernatant before and after enzymatic hydrolysis. Overlay of representative UPLC-FD chromatograms of HEK293^KI ST3GAL5^ supernatant (blue line) and HEK293^KI ST3GAL5^ supernatant after galactosidase and neuraminidase treatment (red line). Retention time 8.8 min corresponds to lactose and 29.3 min to 3’-SL.


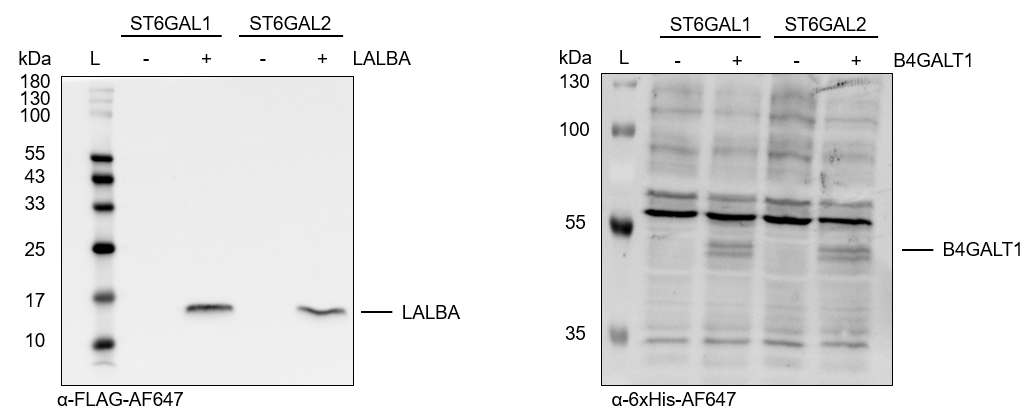


**Supplementary Fig. 19.** Western blot of LALBA/B4GALT1 expression in HEK293^ST6GAL1/2^. Cell lysates from HEK293^KI ST6GAL1/2^ were subjected to SDS-PAGE/western blot 72 hours post-transfection with LALBA-FLAG and B4GALT1-6xHis. Membranes were stained with mouse α-FLAG-AF647 antibody (left) and mouse α-6xHis-AF647 antibody (right), respectively.

**
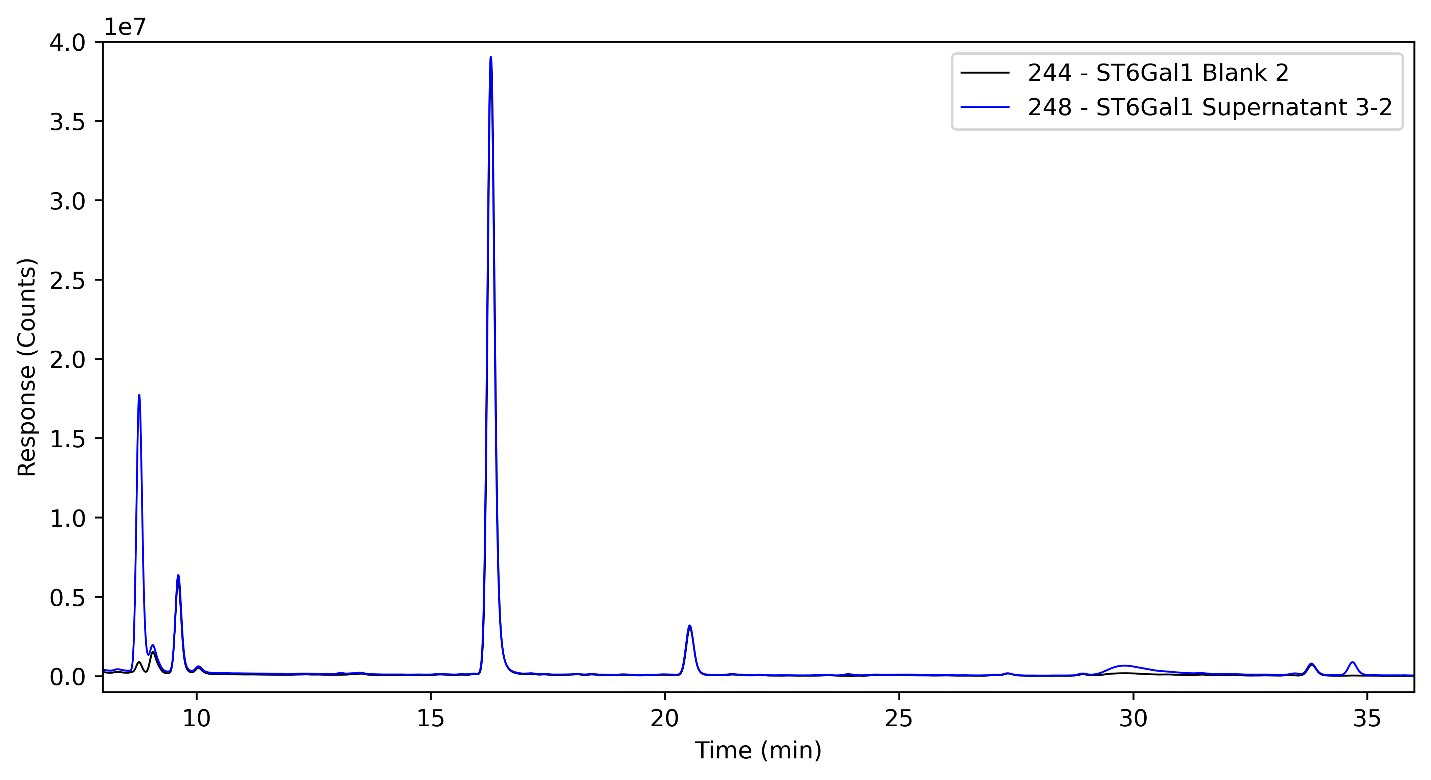
Supplementary Fig. 20.** Chromatograms of mock-transfected and LALBA/B4GALT1-transfected HEK293^ST6GAL2^ supernatant. Overlay of representative UPC-FD chromatograms of HEK293^KI ST6GAL1^ supernatant blank (black line) and HEK293^KI ST6GAL1^ supernatant (blue line). Retention time 8.8 min corresponds to lactose and 34.7 min to 6’-SL.

**
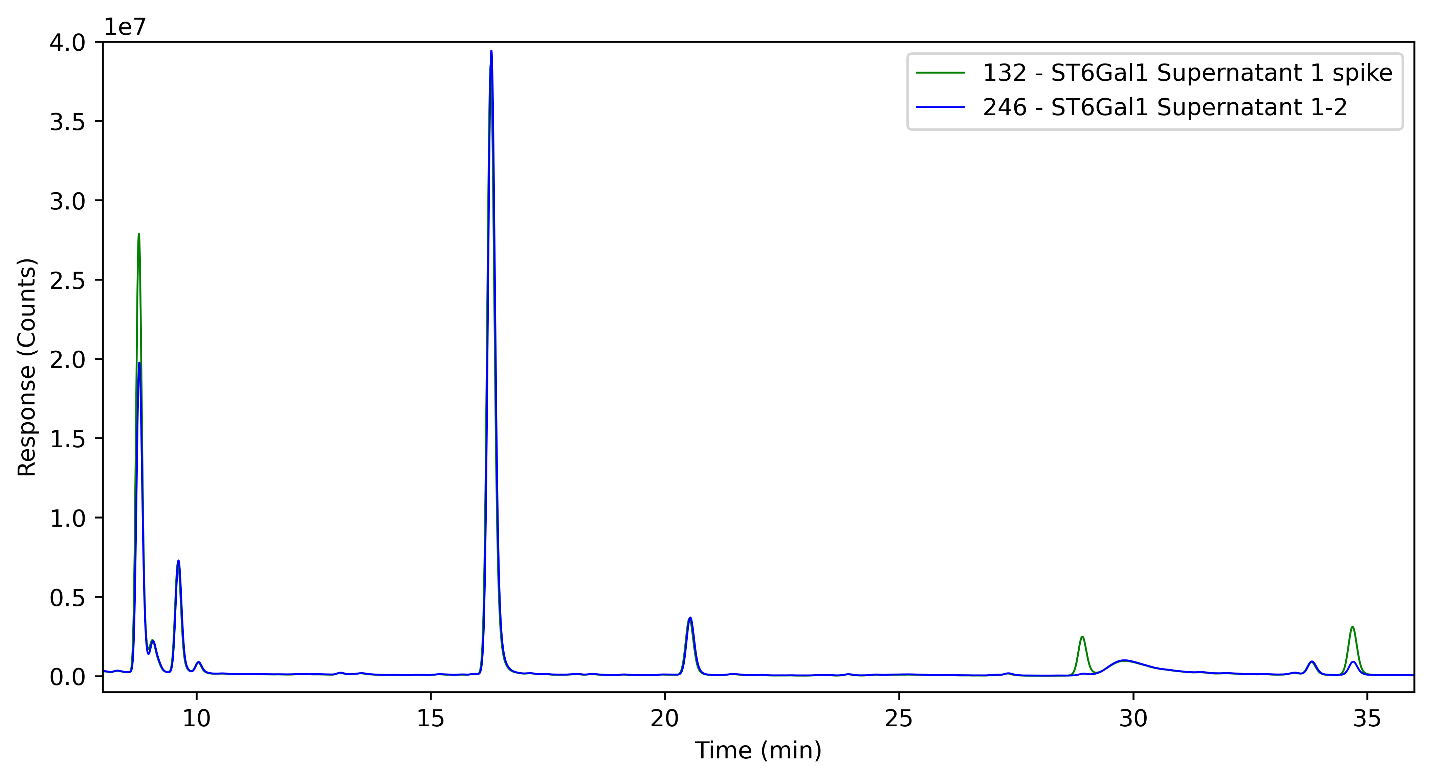
Supplementary Fig. 21.** Chromatograms of LALBA/B4GALT1-transfected HEK293^ST6GAL2^ supernatant spiked with lactose, 3’-sialyllactose, and 6’-sialyllactose. Overlay of representative UPLC-FD chromatograms of HEK293^KI ST6GAL1^ supernatant (blue line) and HEK293^KI ST6GAL1^ supernatant spiked with lactose, 3’-SL and 6’-SL (green line). Retention time 8.8 min corresponds to lactose and 34.7 min to 6’-SL.


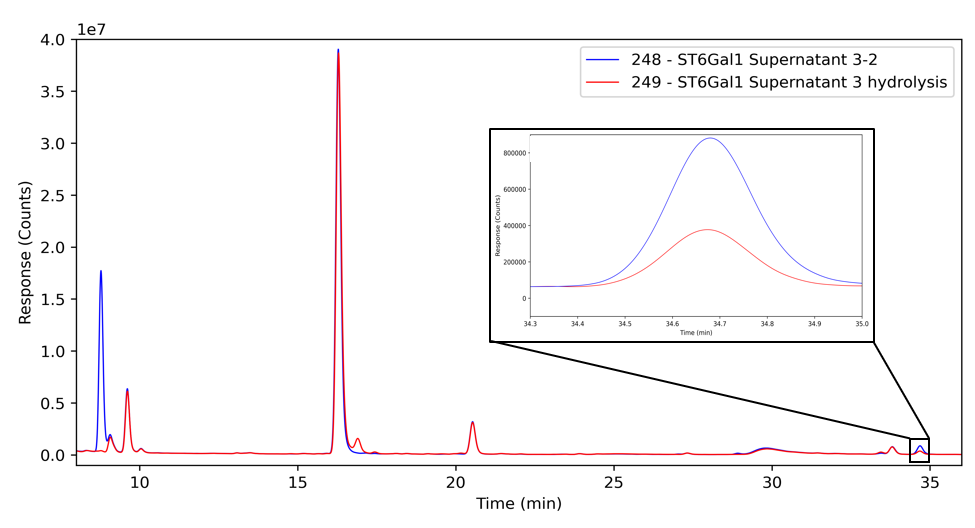
**Supplementary Fig. 22.** Chromatograms of LALBA/B4GALT1-transfected HEK293^ST6GAL2^ supernatant before and after enzymatic hydrolysis. Overlay of representative chromatograms of HEK293^KI ST6GAL1^ supernatant (blue line) and HEK293^KI ST6GAL1^ supernatant after galactosidase and neuraminidase treatment (red line). Retention time 8.8 min corresponds to lactose and 34.7 min to 6’-SL.


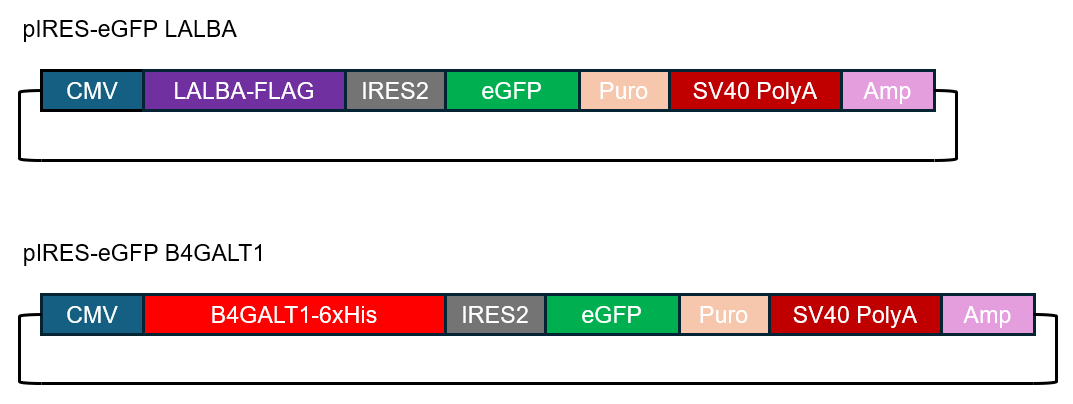
**Supplementary Fig. 23.** Schematic depiction of pIRES-eGFP plasmids from this study encoding LALBA-FLAG (above) or B4GALT1-6xHis (below).

**
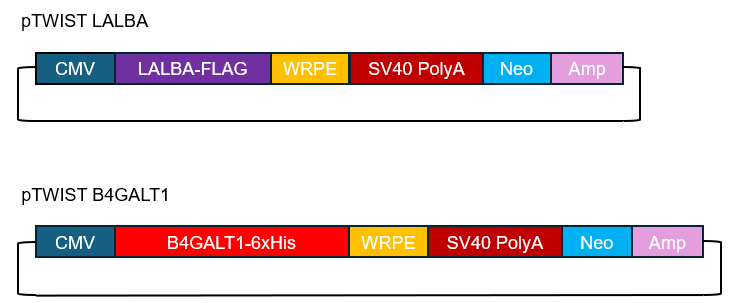
**

**Supplementary Fig. 24.** Schematic depiction of pTWIST plasmids from this study encoding LALBA-FLAG (above) or B4GALT1-6xHis (below).
